# Characterization and Comparative Analysis of the Milk Transcriptome in Two Dairy Sheep Breeds using RNA Sequencing

**DOI:** 10.1038/srep18399

**Published:** 2015-12-18

**Authors:** Aroa Suárez-Vega, Beatriz Gutiérrez-Gil, Christophe Klopp, Christèle Robert-Granie, Gwenola Tosser-Klopp, Juan José Arranz

**Affiliations:** 1 Departamento de Producción Animal, Facultad de Veterinaria, Universidad de León, Campus de Vegazana s/n, León 24071, Spain; 2 INRA, UMR1388 GenPhySE (Génétique, Physiologie et Systèmes d’Elevage), F-31326 Castanet-Tolosan, France; 3 Université de Toulouse, INP, ENSAT, GenPhySE (Génétique, Physiologie et Systèmes d’Elevage), F-31326 Castanet-Tolosan, France; 4 Université de Toulouse, INP, ENVT, GenPhySE (Génétique, Physiologie et Systèmes d’Elevage), F-31076 Toulouse, France; 5 INRA, Sigenae, UR875 Biométrie et Intelligence Artificielle, BP 52627, 31326 Castanet-Tolosan Cedex, France

## Abstract

This study presents a dynamic characterization of the sheep milk transcriptome aiming at achieving a better understanding of the sheep lactating mammary gland. Transcriptome sequencing (RNA-seq) was performed on total RNA extracted from milk somatic cells from ewes on days 10, 50, 120 and 150 after lambing. The experiment was performed in Spanish Churra and Assaf breeds, which differ in their milk production traits. Nearly 67% of the annotated genes in the reference genome (Oar_v3.1) were expressed in ovine milk somatic cells. For the two breeds and across the four lactation stages studied, the most highly expressed genes encoded caseins and whey proteins. We detected 573 differentially expressed genes (DEGs) across lactation points, with the largest differences being found, between day 10 and day 150. Upregulated GO terms at late lactation stages were linked mainly to developmental processes linked to extracellular matrix remodeling. A total of 256 annotated DEGs were detected in the Assaf and Churra comparison. Some genes selectively upregulated in the Churra breed grouped under the endopeptidase and channel activity GO terms. These genes could be related to the higher cheese yield of this breed. Overall, this study provides the first integrated overview on sheep milk gene expression.

Lactation is a specific mammalian function essential for newborn feeding. The milk transcriptome has been characterized in various mammalian species^1^,^2^,^3^. The knowledge of gene expression involved in lactation informs the basic study of mammary gland biology, morphogenesis and metabolic activity as well as enhances our understanding of milk composition in relation to the production of milk-based newborn formulas, milk and milk derivatives.

Livestock species have been used for centuries to provide milk and dairy products for humans. Sheep milk is currently an important source of revenue, ranking fourth in terms of global milk production in 2013 (http://faostat.fao.org/). The dairy sheep industry is primarily concentrated in Europe and the countries near the Mediterranean Sea^4^. Sheep milk is commonly used to produce many cultured dairy products. Spain was the world’s seventh largest producer of sheep milk in 2012 (http://faostat.fao.org/), and the 70% of that milk production is concentrated in the community of Castile and Leon (366.537 million liters in 2012) (http://www.magrama.gob.es/es/). Assaf and Churra are the most important dairy breeds in this region, with 77,896 and 35,094 lactating ewes, respectively, in 2014 (http://www.magrama.gob.es/es/). Churra is a Spanish autochthonous breed, characterized by its rusticity; it is well adapted to the predominantly harsh environments of its production areas. Assaf is a more specialized dairy sheep created as a crossbreed between Awassi (5/8) and Milschchaf (3/8) breeds. Assaf was introduced in Spain in 1977, and its population has increased primarily because of its high production potential and its efficient adaptation to local conditions^5^. Lactation is normalized to 120 days in Churra and 150 days in Assaf. The Assaf milk yield (400 kg) is more than double of the milk yield in Churra (117 kg), although Assaf milk has lower fat (6.65 vs. 7.01) and protein content (5.40 vs. 5.79) (http://www.magrama.gob.es/es/). Hence, Churra milk shows better mature cheese-making yield and organoleptic properties^6^.

Transcriptome sequencing (RNA-seq) technologies provide a unique opportunity to characterize cell transcripts (including alternative splicing and the discovery of new genes and single nucleotide polymorphism in coding sequences), to quantify transcripts and to identify differential regulation in a single experiment^7^. In recent years, RNA-seq technology has been applied to the study of lactating mammary gland in dairy cattle^2^,^8^,^9^.

Various RNA sources have been used to study the mammary gland transcriptome during lactation in mammals, including mammary gland biopsies^3^, milk fat globules^1^ and milk somatic cells (MSCs)^2^. For this study, RNA was extracted from sheep MSCs. The analysis of MSCs is a more accessible method compared to invasive approaches, especially when dynamic studies with several sampling timepoints are required for the same animal^8^. Recent analyses suggest that MSCs are representative sources of RNA in mammary gland tissue, and MSCs isolation is an effective and simple method to study the mammary gland transcriptome through RNA-Seq^10^.

The aim of this research was to gain a better understanding of the sheep lactating mammary gland and to compare the mammary gland transcriptome of two sheep breeds with different dairy production characteristics, Spanish Churra and Assaf. To that end, we performed RNA-seq analysis of MSCs in these two dairy sheep breeds at four different lactation time points with the aim of characterizing the expression pattern of the principal stages of lactation. Here, the successful isolation and generation of global gene expression data from ovine MSCs is reported for the first time. The dynamic characterization of milk sheep expression profile and differences between the milk transcriptomes of Assaf and Churra breeds are described. The results described herein provide a significant advance in our knowledge of sheep lactating mammary gland gene expression and valuable information for future studies.

## Material and Methods

### Animals and sampling

A total of eight healthy sheep were selected to be included in the experiment, four Assaf and four Churra ewes. The animals belong to the commercial farm of the University of León. These sheep were kept in free stall housing, fed with the same rations and had no water restrictions. Animals were milked twice a day: at 8 a.m. and 6 p.m. The lambing by these sheep was between the November 11, 2012, and December 11, 2012. Milk samples were collected on days 10 (D10), 50 (D50), 120 (D120) and 150 (D150) after lambing. The sampling points were established to cover the different physiological stages of the mammary gland across the complete lactation. D10 after lambing is the first day of lactation that is considered totally free of colostrum; it is also the day considered in the normalized lactation for both breeds starts. D50 is a time point close to the lactation peak. The sampling days D120 and D150 correspond to final normalized lactation in Churra and Assaf, respectively. Hence, whereas for Churra D120 is close to final lactation point, for Assaf this time point corresponds to a transition stage form the lactation peak to the final lactation point.

With the aim of maximizing the number of somatic cells present in milk, the sample collection was made one hour after the 8 a.m. routine milking and ten minutes after the injection of 5 IU of OxitocineFacilpart (Syva, León, Spain), as indicated by^11^.All protocols involving animals were approved by the Animal Welfare Committee of the University of Leon, Spain, following proceedings described in Spanish and EU legislations (Law 32/2007, R.D. 1201/2005, and Council Directive 2010/63/EU). All animals used in this study were handled in strict accordance with good clinical practices and all efforts were made to minimize suffering.

To ensure RNA purification of high yield and quality, we used the following protocol during the sampling process. Before sampling, the collecting milk containers were cleaned with RNaseZap (Ambion, Austin, TX, USA) and autoclaved. In the farm, udder cleaning was performed with special care: first, the udders were cleaned with water and soap; then, they were disinfected with povidone iodine; and finally the nipples were cleaned with RNAseZap (Ambion, Austin, TX, USA). Sterile gauze was used to cover the collecting container during milk collection. The milk was passed from the collecting container to RNAse-free 50 ml tubes after collection. Samples were maintained at 4 °C during their transport from the farm to the laboratory.

### RNA extraction

Approximately 50 ml of milk was used for RNA extraction. The pellet of MSCs was obtained as described by^2^ with some modifications. The cells were pelleted by centrifugation at 540 *g* in 50 ml RNAse free sterile tubes for 10 minutes at 4 °C in the presence of a final concentration of 0.5 mM of EDTA. After centrifugation, the supernatant was discarded. During this step, a fatty layer was usually placed in the top of the tube that was removed using a sterile pipette tip. Then, the cell pellet was washed in PBS (pH 7.2) with 0.5 mM EDTA and centrifuged at 540 *g* in 15 ml RNAse free sterile tubes for 10 minutes at 4 °C. The last step was repeated until the fatty layer was minimized. Once the pellet was clean, it was resuspended in 500 μl of Trizol (Invitrogen, Carlsbad, CA, USA) and homogenized by a vortex. RNA extraction continued following a standard Trizol protocol (Invitrogen, Carlsbad, CA, USA).

### RNA sequencing

The Agilent 2100 Bioanalyzer device (Agilent Technologies, Santa Clara, CA, USA) was used to assess the integrity of the RNA. The RNA integrity value (RIN) of the samples ranged between 7.1 and 9. Paired-end libraries with fragments of 300 bp were prepared using the True-Seq RNA-Seq sample preparation Kit v2 (Illumina, San Diego, CA, USA). The fragments were sequenced on an Illumina Hi-Seq 2000 sequencer (Fasteris SA, Plan-les-Ouates, Switzerland), according to the manufacturer’s instructions at CNAG (Centro Nacional de Análisis Genómico, Barcelona, Spain), generating paired-end reads of 75 bp.

### Quality Control, Mapping and Quantification

FastQC (http://www.bioinformatics.babraham.ac.uk/projects/fastqc/) was used to assess the quality of raw sequencing data. Reads were mapped against the ovine genome assembly v.3.1. (Oar_v3.1) using STAR aligner (v.2.3.1y)^12^. The alignments were sorted with Samtools^13^. The data were also tested for contamination using BWA^14^ on the *Escherichia coli* genome.

To compare expression between genes within the same sample, gene expression was estimated using Cuffquant and Cuffnorm packages from Cufflinks^15^. Gene abundances were normalized by library and gene length by calculating Fragments Per Kilobase Of Exon Per Million Fragments Mapped (FPKM) using the Ensembl annotated genes (Oar_v3.1) as a reference.

### Assembly and differential expression analysis

The aim of the assembly was producing a new annotation reference including novel genes and transcripts, to be used in the downstream analysis. The Samtools package was used to remove the duplicated reads and to merge the alignments. The Cufflinks and Cuffmerge tools from the Cufflinks package^15^ were used to create a “transcripts.gtf” file to be used as reference in our assembly. The Cufflinks option “–g” followed by the available gtf file from the Oar_v3.1 reference sequence was used to guide the assembly but not excluding new genes. Cuffmerge was used to filter genes with low or no expression from our reference gtf file. Cuffcompare was used to compare the reference gtf file obtained with Cuffmerge with the reference annotation file downloaded from Ensembl (http://www.ensembl.org/Ovis_aries/Info/Index) to estimate the abundance of transcripts classified in different Cufflinks class codes.

To compare the expression levels of genes across samples, raw counts for the genes and transcripts were obtained using SigCufflinks (available at http://www.sigenae.org). SigCufflinks is a modified version of the cufflinks code that provides raw read counts per gene and transcript, by using the sorted bam file from the alignment and the reference gtf file created in the assembly. The “–G” option of sigcufflinks was used to guide the alignment but excluding new genes.

To evaluate the differentially expressed genes (DEGs) from the RNA-seq data, two different R packages, DESeq2^16^ and edgeR^17^, were used. The DESeq2 package performs independent filtering. In general, genes that have very low counts are not likely to see significant differences due to high dispersion. The edgeR package has no function for independent filtering. To choose a cut-off to eliminate the lowest expressed genes, we plotted a histogram on R with the log_10_ (effmax + 1), thereby maintaining those genes for which the distribution becomes normal and eliminating the genes with less than 15 counts.

RNA-Seq read counts were modeled by a generalized linear model considering the experimental design, with two breed groups (Churra and Assaf) and four lactation time points per group (D10, D50, D120 and D150). The model for both programs includes *breed* and *day* factors and the interaction *day x breed*. After discarding a possible interaction between these two factors, we performed the following analyses with edgeR and DESeq2. First, with the aim of providing a dynamic expression profile of the mammary gland across lactation, differential expression analyses were performed for all the possible time point pairs, considering the Churra and Assaf datasets jointly. Second, to assess the impact of the breed factor on the global expression pattern of sheep lactation, a differential expression analysis between Churra and Assaf, was performed.

DESeq2 and edgeR perform pairwise comparisons between two or more groups using parametric tests where read-counts follow a negative binomial distribution with a gene-specific dispersion parameter. These packages mainly differ in the estimation of the dispersion parameter and the type of normalization they follow. DESeq2 bases the estimation of the dispersion on calculated mean–variance relationships in the data set provided, while edgeR assumes a common dispersion for all the genes. These programs normalize the read count per gene based on total gene depth per individual. Our choice of these two methods was based on the literature evidence for their robustness.

For DESeq2, the DEGs were defined as those genes that had and an absolute log2-fold change >1.5 and an adjusted p-value (*p*_*adj*_-value) < 0.05, whereas edgeR DEGs were those that had a log2-fold change >1.5 and an adjusted FDR (false discovery rate) <0.05. To identify the core DEGs, genes identified as DEGs by the two packages were defined as core DEGs and were subjected to subsequent functional analyses.

### Gene functional classification: GO term enrichment analysis

The web-based Gene Set Analysis Toolkit (WebGestalt^18^) was used to perform a Gene-Ontology (GO) enrichment analysis. Enriched terms were considered statistically significant when *p*_*adj*_-value was <0.05 and a minimum of six genes were grouped for each significant term. The GO terms were categorized in three major functional groups: biological process, molecular function and cellular component.

## Results and Discussion

### Summary statistics for the RNA-seq data

Based on the quality scores of the extracted RNA samples for each breed, samples from four animals were sequenced for time points D10, D50 and D150,and three biological replicates were sequenced for D120. A total of 1,116 million paired-end reads were obtained from the transcriptome sequencing of the 30 milk samples analyzed. Alignment of the reads to the *Ovis aries* genome Oar_v3.1 genome build yielded a mean of 985.05 million reads (88.10%) that aligned to unique locations in the ovine genome per RNA-seq sample; a mean of 1.47 million reads (4.01%) per sample that aligned to multiple locations in the genome; and a mean of 2.84 million reads (7.65%) per sample that did not align to any genome location. No contamination was found in the alignment against the *Escherichia coli* genome. The average percentage of uniquely mapped reads for each sample was substantially higher than that obtained in the analysis of the bovine milk transcriptome, where approximately 65% of the total reads uniquely mapped to the Btau 4.0 reference genome^2^.

The distribution of uniquely mapped reads to annotated genes in the Oar_v3.1 reference is described in Table 1. According to the FPKM value, mapped genes were divided into a low expression group (<10 FPKM), a moderate expression group (≥10 FPKM to 500 FPKM) and a high expression group (≥500 FPKM). Using a threshold of >0.01 FPKM to define potentially significant gene expression^19^, we found an average of 16,757.25 and 16,897.00 unique expressed genes in Assaf and Churra, respectively. These figures represent 66.51% and 67.06%, respectively, of the total annotated genes in the Oar_v3.1 assembly. For both breeds, the lactation time point with the highest number of expressed genes was D50 (17,386 genes in Assaf and 17,192 genes in Churra).From a global view, the transcriptome results revealed that the majority of the genes had low expression (FPKM < 10) across lactation for both breeds (Table 1). The average gene expression levels reported here for sheep MSCs are in agreement with previous reports on the mammary gland transcriptome of other ruminant species^2^,^20^.

A total of 107,877 transcripts were assembled by Cufflinks using the 30 analyzed milk samples. According to the Cuffcompare results (Table 2), 23.95% of the transcripts matched exactly with annotated exons, and a total of 12,057 (11.18%) potentially novel isoforms were predicted. A high percentage, 61.87% (66,739) of transcripts, were annotated as intergenic transcripts, which is in agreement with the results reported from the RNA-Seq analysis of the ovine muscle transcriptome in which the proportion of intergenic fragments ranged between 55.44% (Small-tailed Han sheep) and 66.40% (Dorper sheep) (DP) (using the ovine genome Oarv2.0)^21^. These results unfortunately underline the incompleteness of the annotation of the sheep transcriptome, and the need for further studies to decipher its complexity. The Functional Annotation of Animal Genomes (FAANG) project (http://www.faang.org/) aims to identify all functional elements in animal genomes, and sheep is one of the target species.

### Analysis of the ten most expressed genes in each stage of lactation

**Figure 1:**
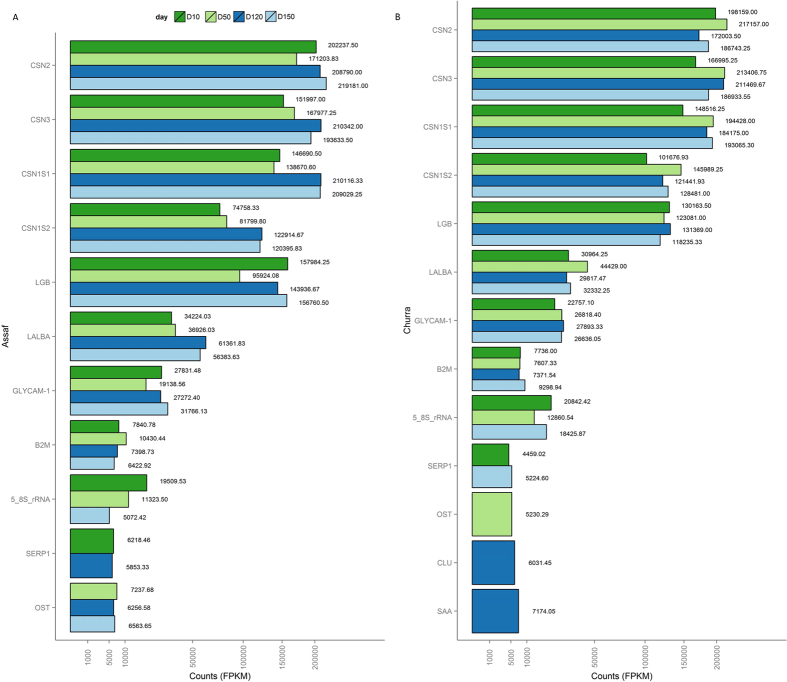
Bar graph with the highly expressed genes in milk somatic cells at 10, 50, 120 and 150 days of lactation. FPKM values are represented in the X-axis, whereas the gene names are indicated in Y-axis. A color code is used to represent the four time points studied. (**a**) Top-10 highly expressed genes in Assaf. (**b**) Top-10 highly expressed genes in Churra.

Gene abundances were normalized by library size and gene length (FPKM) to determine the list of the most expressed genes at each lactation time point. This normalization approach facilitates the comparison of genes within a sample^22^. Table 1 shows the number of genes with the highest FPKM (≥500) for each breed at each time point analyzed. The results show that in sheep milk, the top-10 genes have very high expression values. For both breeds, Churra and Assaf, the ten highest expressed genes at each time point accumulate at approximately 70% of the total gene FPKM reads, which means that a small number of genes contribute to a large fraction of the total RNA extracted from MSCs. A total of 13 genes, ranging between means of 4,459 to 219,181 FPKM, are encompassed in the top-10 highest expressed genes (Fig. 1). In general, with exceptions for very specific changes in the profile expression, the same genes are highly expressed at all of the different time points assessed during sheep lactation. In contrast, in dairy cattle late lactation, the caseins are not included in the top-expressed category and are instead replaced by other genes involved in proteolysis, anti-apoptotic activity and immune functions^2^.

The genes with the highest FPKM values for both breeds and at the four studied lactation time points are *CSN2* (β-casein), *CSN3* (κ-casein), *ENSOARG00000005099* (*LGB*, β-lactoglobulin), *CSN1S2* (casein-α-S2), *CSN1S1* (α-S1-casein) and *LALBA* (α-lactoalbumin). These highly expressed genes encode four caseins and two whey proteins, principal components of milk. Caseins and whey proteins encompass the 5.5% of total milk composition in sheep, a higher percentage than in bovine milk (3.2%)^23^. Caseins and whey protein expression remained constant for Churra along lactation. As shown in Fig. 1, these six genes were slightly more abundant in Churra sheep at the first two sampling time points (D10 and D50) than in Assaf sheep. In contrast, the expression of caseins and whey proteins in the Assaf breed underwent an increase at the last two time points studied (D120 and D150). The *LALBA* gene, which encodes for the α-lactoalbumin, had higher expression in Assaf at D120 and D150. A SNP described in this gene has been associated with a higher milk protein and fat content (milk concentration) in Churra sheep^24^. The α-lactoalbumin is a major milk whey protein involved in the synthesis of lactose, which is responsible for drawing water into the milk. The higher milk yields of Assaf as compared to Churra could be due, among other factors, to higher expression levels of the *LALBA* gene in this breed.

Apart from caseins and whey proteins, there are two other highly expressed genes in the two breeds and at the four stages of lactation studied: *GLYCAM-1* and *B2M*. *GLYCAM-1* encodes the glycosylation-dependent cell adhesion molecule 1, a member of the glycoprotein mucin family, which is a component of the milk fat globule membrane^25^ and appears to be hormonally regulated in the sheep mammary gland^26^. *B2M* encodes for the beta-2-microglobulin protein, an integral component of the Fc receptor heterodimer, which is involved in transferring Immunoglobulin G (IgG) from serum into milk across mammary epithelial cells^27^. Some *B2M* haplotypes have been reported to be related to higher concentrations of IgG1 in bovine milk^28^. Increasing IgG levels in milk could become important as IgG enhanced dairy products are in demand by consumers to obtain protective immunity^29^.

Three other genes included among the top-10 highly expressed genes were found in the two studied breeds but not across all the time points. The *5.8S rRNA* (5 8S ribosomal RNA) gene is a non-coding RNA component of the large subunit of the eukaryotic ribosome. It is part of the ten most expressed genes in MSCs at D10, D50 and D150 for Churra and Assaf breeds. Expression of this gene in the Churra breed remains constant, while in the Assaf breed it decreases as lactation proceeds (Fig. 1). In the rabbit mammary gland, expression of rRNA is related to high levels of protein synthesis, correlations between prolactin binding to mammary epithelial cells and high levels of rRNA synthesis^30^. The *SERP1* gene, which encodes the stress-associated endoplasmic reticulum protein 1, is among the ten most highly expressed genes at D10 in both breeds, at D120 in Assaf and at D150 in Churra. This protein is enhanced by stress causing the accumulation of unfolded proteins in endoplasmic reticulum, suppressing the aggregation and/or degradation of newly synthesized integral membrane proteins, and facilitating their glycosylation when the stress is removed^31^. The endoplasmic reticulum stress gene network has been analyzed in the mammary glands of ruminants and non-ruminants to study their role in maintaining protein cellular homeostasis as well as the potential implications in lipid homeostasis^32^. The *OST* (also known as *SPP1*) gene encodes for osteopontin, a major phosphoglycoprotein highly expressed in the mammary gland. This gene is one of the ten most expressed genes at D50, D120 and D150 of lactation in the Assaf breed but only at D50 in the Churra breed. The role of osteopontin in the mammary gland is not completely clear. Several studies have shown an association between the expression of the *OST* gene and milk yield by enhancing the expression of caseins *CSN2* and *CSN3*^33^, and *OST* has also been related to mammary gland morphogenesis^34^ and new born immunity^35^.

In addition, the top-10 list of genes at D120 in Churra sheep contained two genes that were not included in this category in the Assaf breed at any time point. One of them, *CLU*, encodes for the clusterin protein. In mice, the *CLU* gene is highly expressed during mammary gland prepartum development and also at the involution stage and is highly associated with tubuloalveolar morphogenesis and alveolar epithelial cell differentiation^36^. Here, in agreement with the observations described in mice, *CLU* is highly expressed at the end of lactation. In Assaf sheep, where the lactation is longer than in Churra sheep, although this gene was not found within the top-10 list of any time point, it was found within the group of highly expressed genes at D120 and D150. *CLU* has also been found to be associated to apoptotic events and environmental stress^36^. In normalized lactation, D120 corresponds to the last stage lactation in Churra sheep; hence, the intense remodeling of mammary epithelial cells due to the milking on this last stage could stimulate the *CLU* expression.The second gene exclusively identified in Churra sheep in the top-10 list at D120 is *SAA*, which encodes for a member of the serum amyloid family of apolipoproteins. High levels of *SAA* have been related to states of inflammation in the mammary gland^37^. However, studies in pigs in healthy lactating mammary glands showed an increase of this gene’s mRNA in the second half of lactation, providing evidence that this peptide could play a physiological role in the normal mammary gland, stimulating neonatal gut immune responses^38^.

### Differentially expressed genes across lactation

**Figure 2:**
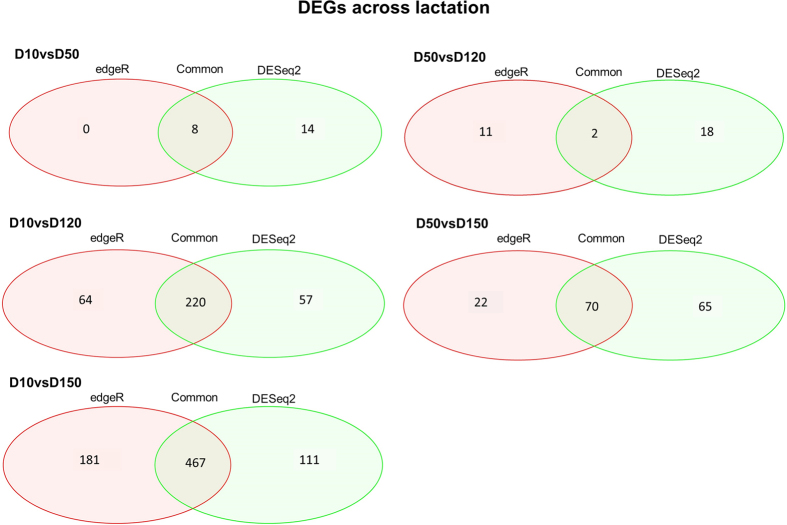
Venn diagrams showing the number of differential expressed genes (DEG) across lactation (considering the four time points studied) in sheep. For all the time point pair comparisons considered, and considering the two breed datasets jointly, the number of DEG identified by the edgeR analysis (red ellipse), the DESeq2 analysis (green ellipse) or both programs (intersection) is indicated.

**Figure 3:**
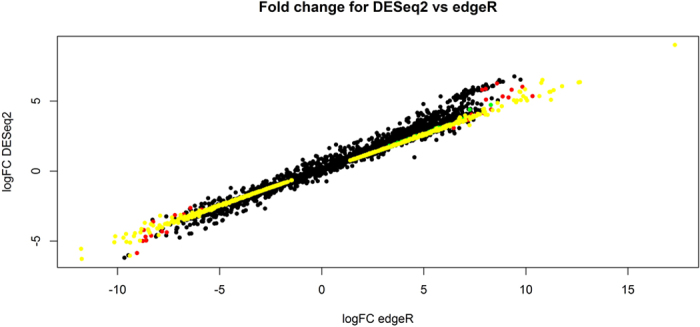
Comparison of differentially expressed genes (DEGs) detected with DESeq2 and edgeR in the D10 *vs.* D150 contrast. (**a**) Comparative graphical representation of the logFC values resulting from the differential expression analysis performed for the D10 *vs.* D150 contrast with the edgeR package (X-axis) and the DESeq2 package (Y-axis). To help the comparison, a color code is used to distinguish the following categories: logFC values of the genes exclusively detected as DEGs by edgeR (red); logFC values of the genes exclusively detected as DEGs by DESeq2 (green); logFC values of the genes detected as DEGs by both packages (yellow); logFC values of the genes not identified as DEG by any software (black). For the genes detected as DEGs by both packages (colored in yellow) the expression levels (logFC values) measured by the two packages showed a high and linear correlation (*r*^*2*^ = 0.99).

For the eight time points comparisons performed, the number of unique DEGs identified was 2,585 with edgeR and 751 with DESeq2. A total of 573 DEGs, of which 325 were annotated genes, were detected in common by the two packages. The number of DEGs identified in each specific comparison is represented in Fig. 2 (all except the D120 vs. D150 comparison which did not show any DEGs). The comparison D10 vs. D150, which showed the highest number of DEGs (648 with edgeR and 578 with DESeq2), was used to compare the significant results from the two packages (Fig. 3). When considering the genes commonly identified by the two software solutions, we observed a strong linear correlation between the logFC values obtained by the two packages (*r*^*2*^ = 0.99). Based on this, only the annotated DEGs commonly identified with the two packages whose number ranged between 2 (D50 *vs.*D120 contrast) and 247 (D10 *vs.*D150 contrast) were subjected to a functional GO term enrichment analysis.

**Figure 4:**
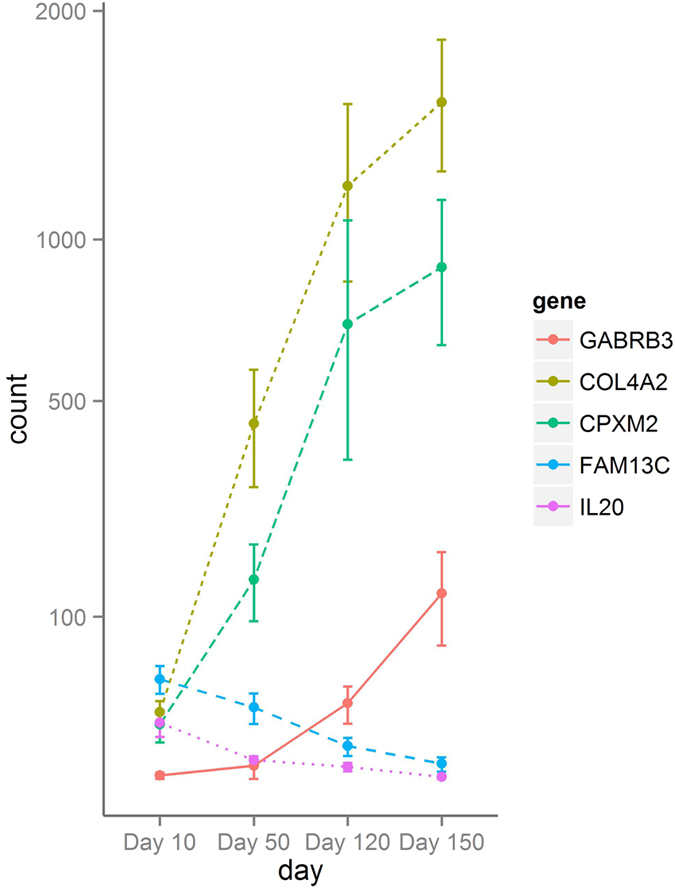
Expression profile of the five differentially expressed genes (DEGs) showing the largest fold-changes across lactation. The counts normalized by sequencing depth with the DESeq2 package (y-axis) are represented against the lactation stages studied in the present work (x-axis). For each gene at each stage of lactation the point indicates the mean of counts over all samples at this stage and error bars show the standard error of the mean.

The five genes showing the largest changes in fold-changes across lactation is given in Supplementary Table S1 and represented in Fig. 4. As it can be observed, the genes *GABRB3, COL4A2* and *CPXM2* showed a gradual and pronounced increase from the beginning to the end of lactation (with 17.29, 12.64 and 12.58 logFC increase in D150 compared to D10), whereas the expression of *FAM13C* and *IL20* follows the inverse profile (with -11.78 and -11.75 logFC decrease in D150 compared to D10). *GABRB3* encodes for the gamma-aminobutyric acid receptor subunit beta-3, and in a study in lactating rats this gene showed changes in the expression level greater than 10-fold^39^, although in that case *GABRB3* was upregulated at the beginning of the lactation^39^. The *COL4A2* gene encodes the alpha-2 chain of type IV collagen. Collagen IV is part of the mammary gland extracellular matrix (ECM), being an essential protein for supporting basal lamina structure and maintaining cell viability by providing an anchor for mammary epithelial cells^40^. In addition, cryptic domains of type IV collagen fragments appear to function as anti-angiogenic factors^41^. Hence, the increment of *COL4A2* expression towards late lactation could be linked to the basal lamina turnover and the vascular regression that takes places during the mammary gland involution. *CPXM2* encodes for carboxypeptidase X (M14 family), member 2. This protein has metallocarboxypeptidase activity and shows an increased expression across lactation. High levels of endopeptidases are related to ECM remodeling, and linked to both, mammary gland development and involution^42^. In relation to the two genes showing a decreasing profile across lactation, *FAM13C* encodes for the Family with sequence similarity 13, member C and its function is presently unknown. Another member of FAM13 family, *FAM13A* has been related to a milk protein QTL in Israeli Holstein cows^43^ and polymorphisms on this gene were related to increased levels of somatic cell counts (SCC) in milk in Jersey cattle^44^. *IL20*, which encodes for Interleukin-20, belongs to the IL10 family cytokine cluster^45^ and its expression has been related to skin, lungs, reproductive organs and various glands^45^. Among other functions, *IL20* promotes angiogenesis^46^. During early lactation, when this gene shows its highest expression levels, the number of venous capillaries surrounding alveoli increases as a result of extensive glandular growth^47^.

In the across lactation study, the significant GO enrichment terms (*p*_*ad*j_ < 0.05), for the annotated DEGs were categorized into 140 functional groups (Supplementary Table S2), across the three GO established categories: “biological processes” (106 terms), “cellular component” (30 terms) and “molecular function” (4 terms). Interestingly, the most significant terms of these three categories were associated to DEGs identified in the D10 *vs* D150 comparison.

**Figure 5:**
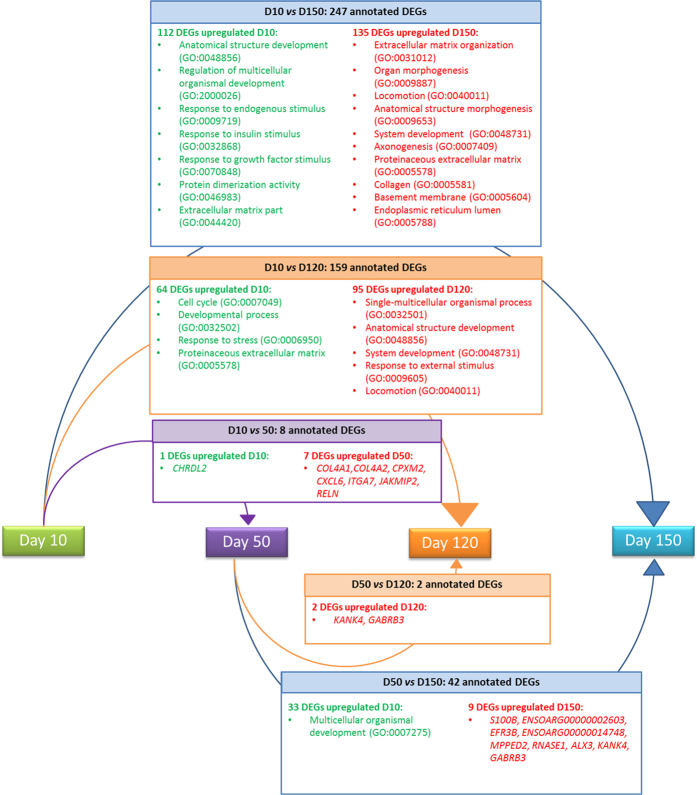
Schematic representation of the most representative enrichment GO terms highlighted by our functional analysis in relation to the DEG identified across lactation. The four lactation time points considered are indicated with colored boxes: day 10 (D10, green), day 50 (D50, purple), day 120 (D120, orange) and day 150 (D150, blue). The different arrows correspond to a specific time-point comparison and are linked to a summary box where the number of annotated upregulated DEGs in the two time-points compared are shown. The most representative GO terms associated to the identified DEGs are also indicated. Note that if no significant results were identified in the GO enrichment analysis the upregulated genes at the corresponding stage are indicated.

Due to the large number of results generated in the GO enrichment analysis for all the comparisons, a general picture of the most representative enrichment terms related to the expression changes taking place across lactation is given in Fig. 5. Lactation maintenance in mammals results from the balance between competing processes of mammary development, prolactin signaling and involution pathways^48^. In relation to these processes, several of the enriched terms found in our RNA-Seq across lactation analysis are related to developmental processes (GO:0032502). Referring to involution, this mechanism produces changes in mammary gland architecture, including ECM remodeling, collapse of alveoli and differentiation of adipocytes^48^. At final lactation (D150), terms related to cellular components of ECM are enriched (proteinaceous extracellular matrix (GO:0005578 ), collagen (GO:0005581) and basement membrane (GO:0005604)). These terms grouped genes that belong to mammary gland ECM (*COL4A1*, *COL4A2*, *COL18A1, COL5A2, COL21A1*, *DCN*, *HSPG2*, *TNC* and *SPARC*) and have been associated with the onset of mammary gland involution^40^. Other genes upregulated at D150 in relation to the ECM-receptor interaction were *RELN* and *OST*, which are involved in the regulation of the mammary gland morphogenesis^34^,^49^. These results reflect the unique attributes of the mammary gland as a dynamic organ, in continuous development across its several distinct functional states. Regarding the other competing process occurring in the mammary gland, our results did not show a direct relationship with prolactin signaling. However, for the upregulated DEGs at D10 we found as significant the GO terms response to insulin stimulus (GO:0032868) and response to grow factor stimulus (GO:0070848). It is remarkable that both, insulin and growth hormone are known to increase prolactin lactogenic effect^49^. Additionally, it is worthy to highlight the significant GO term related to endoplasmic reticulum lumen (GO:0005788) in upregulated DEGs at D150 of lactation. This organelle is linked to the lipid secretor mechanism of the mammary epithelial cells^50^. Initial analyses on the expression of genes related to the mammary gland fat metabolism suggest that genes implicated in the *de novo* synthesis of sheep milk fat show increased expression at late lactation^51^.

As stated, major differences in gene expression levels and DEGs in sheep were found between extreme stages of lactation. These observations differ with those reported in cattle, where the analysis of the MSC transcriptome showed that the highest number of DEGs was found between the transition and the peak lactation stages^2^. To interpret these differences, we have to take into account that dairy cattle have a longer lactation (normalized to 305 days in Holstein) than sheep (normalized to 150 days in Assaf and 120 days in Churra).

### Differentially expressed genes between Churra and Assaf

In the comparison between the Churra and Assaf breeds, edgeR identified a total of 1,039 DEGs, whereas 774 genes were identified as DEGs with the DESeq2 package. A total of 630 genes, of which 256 were annotated, were found in common as DEGs from both packages. A total of 172 annotated genes were upregulated in the Churra breed, and 84 annotated genes were upregulated in the Assaf breed.

In the comparison between breeds, the significant (*p*_*ad*j_ < 0.05) GO enrichment terms for the annotated DEGs were categorized into 46 functional groups (Supplementary Table S3). Twenty terms were significantly enriched in relation to the “biological processes” category. The most significantly enriched GO terms within this dataset were immune response (GO: 0006955), and immune system process (GO:0002376), which were associated to upregulated genes in the Assaf breed. In the “cellular component” GO term category, 11 terms were significantly enriched; the most significant ones were related to extracellular region (GO: 0005576) and extracellular region part (GO:0044421) also associated to Assaf upregulated genes. The rest of significantly enriched terms (11) belonged to the “molecular function” category, and, the most significant ones were receptor binding (GO:0005102) related to Assaf DEGs, and three terms related to serine hydrolase (GO:0017171) and endopeptidase activities (GO:0008236, GO:0004252), the three of them related to Churra DEGs.

Based on the findings in the functional GO enrichment analysis, gene expression in the Assaf breed is shown to be largely enriched by genes involved in immune (GO:0006955) and stress (GO:0006950) responses. Immune response processes in milk are related to increments in SCC. Subclinical infections elicit elevated SCC but other variables like breed have been shown to influence on the levels of milk SCC^6^. In absence of mastitis Assaf breed has been shown to have higher average levels of SCC than Churra sheep^52^. Other studies have shown a relationship between milk SCC levels and cheese sensory characteristics (hardness, intensity of taste, and pungency)^6^, which could be explained by the action of immune related peptides in cheese manufacturing.

The GO terms related to endopeptidase activity (GO: 0004175, GO:0004252), which were associated to DEGs upregulated in Churra sheep, may be of special interest. Endopeptidases have effects on the physicochemical characteristics and quality of dairy products^53^ and have a direct influence on cheese flavor due to the production of short sapid peptides and amino acids during the ripening procedure^54^. The principal indigenous proteinases in milk are plasmin, cathepsins and elastase^54^. However, none of these protein encoding genes were found as DEGs in our analysis. Among the upregulated genes grouped under the endopeptidase activity term, it is worth to highlight that six genes belong to the kallikrein gene family (*KLK5*, *KLK6*, *KLK7*, *KLK10*, *KLK12* and *KLK13*). Kallikreins belong to a group of serine proteases that have trypsin or chymotrypsin activity. Among other physiological functions, these proteins are responsible for the coordination of blood pressure, semen liquefaction and skin desquamation^55^. A study performed by^55^ in human biological fluids confirms the presence of *KLK* genes in milk. Although the plasminogen activator KLK1 has been related with mammary gland involution^49^, the role of these serine proteases in milk has not been determined yet. Additionally, nine DEGs upregulated in the Churra breed were grouped under the significant ion channel activity term (GO:0005216). The ion channel activity has been related to the regulation of milk secretion^56^. Hence, in dairy cattle the secretion of K + , Na + and Cl− determines approximately 40% of the driving force, with the rest being determined by lactose^57^. Moreover, mineral concentrations also affect the milk physicochemical properties, including renneting properties^58^. The differential expression observed between Churra and Assaf breeds for these groups of genes (kallikrein, ion channel related genes) seems noteworthy when considering the differences in milk volume and physicochemical properties influencing renneting and ripening processes.

Overall, this study represents the first integrated overview on the dynamic expression profile of the milk sheep transcriptome. In addition, this study allowed us to compare the milk transcriptome of two dairy sheep breeds, Churra and Assaf, showing that the differences in the gene expression profiles, although small, could serve to identify candidate genes explaining the known differences in production characteristics (milk yield, and milk composition) that exist between these two breeds. This work may provide fundamental information for future studies on specific pathways involved in lactation as well as the functional annotation of novel genes detected.

## Additional Information

**How to cite this article**: Suárez-Vega, A. *et al*. Characterization and Comparative Analysis of the Milk Transcriptome in Two Dairy Sheep Breeds using RNA Sequencing. *Sci. Rep.* **5**, 18399; doi: 10.1038/srep18399 (2015).

## Supplementary Material

Supplementary Information

## Figures and Tables

**Table 1:** RNA-Seq gene expression distribution for the two breeds and the four lactation time points studied in the present work.

	Churra				Assaf			
	D10	D50	D120	D150	D10	D50	D120	D150
Highly expressed genes (≥500 FPKM)	129	109	116	111	129	141	107	100
Medium expressed genes (≥10 FPKM to 500 FPKM)	5982	4747	5238	4986	5977	5916	4664	4397
Lowly expressed genes (<10 FPKM)	10688	12336	11393	11753	10914	11329	11448	11907
Total expressed genes	16799	17192	16747	16850	17020	17386	16219	16404
Non expressed genes	8397	8004	8449	8346	8176	7810	8977	8792

**Table 2:** Classification of the transcripts identified in the mammary gland samples in relation to the Ensembl annotated sheep genes (Oar_v3.1) based on the Cuffcompare tool of Cufflinks.

Cuffcompare_class	Number_transcripts	Percentage
Complete match of intro chain	25833	23.95
Multiple classifications	0	0
Contained in thereference	0	0
Possible pre-mRNA fragment	0	0
Transcript falling within a reference intron	0	0
Potentially novel isoforms	12057	11.18
Generic overlap with a reference transcript	1021	0.95
Possible polymerase run-on fragment	0	0
Intergenic transcript	66739	61.87
Exonic overlap on opposite strand	2166	2.01
Repeat	0	0
Overlapping intron transfrag in the other strand	61	0.06
