# Supplementary material for: Characterization and Comparative Analysis of the Milk Transcriptome in Two Dairy Sheep Breeds using RNA Sequencing
Source: Sci Rep. 2015 Dec 18;5:18399. doi: 10.1038/srep18399 (PMC4683406; doi:10.1038/srep18399)
Supplement: Supplementary Information [file srep18399-s1.pdf]

Characterization and Comparative Analysis of the Milk Transcriptome in Two Dairy Sheep Breeds using RNA Sequencing

Aroa Suárez-Vega[1], Beatriz Gutiérrez-Gil[1], Christophe Klopp[5], Christèle Robert-Granie[2,3,4], Gwenola Tosser-Klopp[2,3,4], Juan José Arranz[1]

Supporting Information:

Table S1 | Results for the five genes with the highest fold changes across lactation

Table S2 | Gene Ontology (GO) assignment of the DEGs detected across lactation.

Table S3 | Gene Ontology (GO) assignment of the DEGs detected between breeds (Churra and Assaf).

Table S1. Results for the five genes with the highest fold changes across lactation

Gene_ID	logFC (D10 vs. D50)	FDR (D10 vs. D50)	logFC (D10 vs. D120)	FDR (D10 vs. D120)	logFC (D10 vs. D150)	FDR (D10 vs. D150)	logFC (D50 vs. D120)	FDR (D50 vs. D120)	logFC (D50 vs. D150)	FDR (D50 vs. D150)
GABRB3	2,316331485	0,999962514	12,67984464	2,04336E-07	17,29808779	9,80035E-15	10,36351315	0,000365164	14,9817563	2,36928E-11
COL4A2	9,105600465	5,48654E-08	11,84537387	8,05957E-13	12,64516354	5,64668E-15	2,739773403	0,62308996	3,539563072	0,13059736
CPXM2	7,31119492	0,014127456	11,61179309	3,9737E-07	12,58061621	9,02666E-09	4,300598174	0,478126007	5,269421289	0,070379995
IL20	-4,907331453	0,216703477	-9,341229827	0,001692214	-11,75869166	8,69752E-07	-4,433898375	0,638454838	-6,851360206	0,073433096
FAM13C	-1,778421558	0,747086717	-5,602645329	0,096944154	-11,78677698	4,83168E-06	-3,824223772	0,645459538	-10,00835542	0,001567396

Table S2. Gene Ontology (GO) assignment of the DEGs detected across lactation.

D10 *vs.* D150. Upregulated D10					
**Database**	**Name**	**ID**	**adjP**	**Statistics**	**Genes**
biological process	anatomical structure development	GO:0048856	0.0037	C=4030; O=32; E=16.80; R=1.90; rawP=3.28e-05	*ASPA, PAPSS2, NPTX1, STAB1, GSS, FOXP2, C1QB, NMUR2, C1QC, APOE, CAV2, COL9A2, SOX6, PALMD, FGFR4, KCNQ3, GPC4, PRICKLE1, LAMA2, KL, ROS1, TMOD1, PDZRN3, AHSG, TEX11, CITED1, SOSTDC1, AR, IL20, CHRDL2, IL1RAPL2, SOX5*
biological process	regulation of multicellular organismal development	GO:2000026	0.0037	C=1098; O=15; E=4.58; R=3.28; rawP=3.24e-05	*AHSG, FGFR4, FOXP2, CITED1, ASPA, C1QC, AR, APOE, PRICKLE1, IL20, STAB1, LAMA2, KL, SOX5, SOX6*
biological process	response to endogenous stimulus	GO:0009719	0.0038	C=1004; O=14; E=4.19; R=3.34; rawP=5.04e-05	*AHSG, FGFR4, C1QB, CITED1, APOBEC1, PLA2G1B, RERG, STEAP2, AR, KL, COL16A1, SOX5, SOX6, GSS*
biological process	developmental process	GO:0032502	0.005	C=4572; O=33; E=19.06; R=1.73; rawP=0.0002	*ASPA, PAPSS2, GADD45G, NPTX1, STAB1, GSS, FOXP2, C1QB, NMUR2, C1QC, APOE, CAV2, COL9A2, SOX6, PALMD, FGFR4, KCNQ3, GPC4, PRICKLE1, LAMA2, KL, ROS1, TMOD1, PDZRN3, AHSG, TEX11, CITED1, SOSTDC1, AR, IL20, CHRDL2, IL1RAPL2, SOX5*
biological process	regulation of developmental process	GO:0050793	0.005	C=1436; O=16; E=5.99; R=2.67; rawP=0.0002	*FGFR4, PALMD, ASPA, PRICKLE1, STAB1, LAMA2, KL, FOXP2, AHSG, CITED1, C1QC, APOE, AR, IL20, SOX6, SOX5*
biological process	system development	GO:0048731	0.005	C=3521; O=28; E=14.68; R=1.91; rawP=0.0002	*FGFR4, KCNQ3, ASPA, PAPSS2, NPTX1, PRICKLE1, STAB1, LAMA2, KL, GSS, AHSG, PDZRN3, FOXP2, C1QB, TEX11, CITED1, NMUR2, SOSTDC1, C1QC, AR, APOE, IL20, CHRDL2, IL1RAPL2, CAV2, COL9A2, SOX5, SOX6*
biological process	single-organism process	GO:0044699	0.005	C=7682; O=46; E=32.03; R=1.44; rawP=0.0002	*ASPA, PAPSS2, RERG, GADD45G, NPTX1, PKIG, LIPG, STAB1, MNS1, GSS, FOXP2, C1QB, NMUR2, C1QC, APOE, COL9A2, CAV2, SOX6, CTH, FGFR4, KCNQ3, PRICKLE1, LAMA2, KL, HTR4, ROS1, HYAL4, GAL3ST4, NLRC4, TMOD1, PDZRN3, AHSG, TEX11, CITED1, SOSTDC1, C1QA, PLA2G1B, ABCG8, AR, IL20, SYT4, CHRDL2, IL1RAPL2, COL16A1. LPAR4. SOX5*
biological process	regulation of multicellular organismal process	GO:0051239	0.005	C=1772; O=18; E=7.39; R=2.44; rawP=0.0002	*FGFR4, ASPA, PRICKLE1, LIPG, STAB1, LAMA2, KL, FOXP2, AHSG, NMUR2, CITED1, C1QC, ABCG8, APOE, AR, IL20, SOX5, SOX6*
biological process	response to insulin stimulus	GO:0032868	0.005	C=265; O=7; E=1.10; R=6.34; rawP=0.0001	*AR, AHSG, FGFR4, CITED1, APOBEC1, KL, PLA2G1B*
biological process	multicellular organismal process	GO:0032501	0.009	C=5644; O=37; E=23.53; R=1.57; rawP=0.0004	*ASPA, PAPSS2, GADD45G, NPTX1, LIPG, STAB1, GSS, FOXP2, C1QB, NMUR2, C1QC, APOE, CAV2, COL9A2, SOX6, FGFR4, KCNQ3, PRICKLE1, LAMA2, KL, ROS1, NLRC4, TMOD1, PDZRN3, AHSG, TEX11, CITED1, SOSTDC1, PLA2G1B, ABCG8, AR, IL20, SYT4, CHRDL2, IL1RAPL2, COL16A1. SOX5*
biological process	central nervous system development	GO:0007417	0.0103	C=688; O=10; E=2.87; R=3.49; rawP=0.0005	*AHSG, C1QB, FOXP2, CITED1, NMUR2, ASPA, NPTX1, IL1RAPL2, SOX5, SOX6*
biological process	multicellular organismal development	GO:0007275	0.0129	C=4077; O=29; E=17.00; R=1.71; rawP=0.0008	*FGFR4, KCNQ3, ASPA, PAPSS2, GADD45G, NPTX1, PRICKLE1, STAB1, LAMA2, KL, GSS, AHSG, PDZRN3, FOXP2, C1QB, TEX11, CITED1, NMUR2, SOSTDC1, C1QC, AR, APOE, IL20, CHRDL2, IL1RAPL2, CAV2, COL9A2, SOX5, SOX6*
biological process	single-multicellular organism process	GO:0044707	0.0129	C=5612; O=36; E=23.40; R=1.54; rawP=0.0008	*ASPA, PAPSS2, GADD45G, NPTX1, LIPG, STAB1, GSS, FOXP2, C1QB, NMUR2, C1QC, APOE, CAV2, COL9A2, SOX6, FGFR4, KCNQ3, PRICKLE1, LAMA2, KL, ROS1, NLRC4, TMOD1, PDZRN3, AHSG, TEX11, CITED1, SOSTDC1, PLA2G1B, ABCG8, AR, IL20, SYT4, CHRDL2, IL1RAPL2, SOX5*
biological process	response to hormone stimulus	GO:0009725	0.0129	C=723; O=10; E=3.01; R=3.32; rawP=0.0007	*AHSG, FGFR4, C1QB, CITED1, APOBEC1, PLA2G1B, RERG, STEAP2, AR, KL*
biological process	response to chemical stimulus	GO:0042221	0.0166	C=2746; O=22; E=11.45; R=1.92; rawP=0.0011	*FGFR4, KCNQ3, PAPSS2, RERG, LIPG, LAMA2, KL, GSS, C1QB, AHSG, CITED1, APOBEC1, PLA2G1B, STEAP2, AR, APOE, COL16A1, COL9A2, SOX5, CYBRD1, SOX6, CTH*
biological process	nervous system development	GO:0007399	0.0169	C=1724; O=16; E=7.19; R=2.23; rawP=0.0015	*KCNQ3, ASPA, NPTX1, PRICKLE1, LAMA2, GSS, FOXP2, C1QB, AHSG, NMUR2, CITED1, APOE, COL9A2, IL1RAPL2, SOX6, SOX5*
biological process	positive regulation of response to stimulus	GO:0048584	0.0169	C=1084; O=12; E=4.52; R=2.66; rawP=0.0015	*FGFR4, C1QB, CITED1, C1QA, PLA2G1B, C1QC, AR, IL20, KL, CAV2, CTH, NLRC4*
biological process	response to growth factor stimulus	GO:0070848	0.0169	C=297; O=6; E=1.24; R=4.85; rawP=0.0014	*FGFR4, APOE, CITED1, KL, SOX5, SOX6*
biological process	response to organic nitrogen	GO:0010243	0.0169	C=655; O=9; E=2.73; R=3.30; rawP=0.0015	*AHSG, FGFR4, CITED1, APOBEC1, PLA2G1B, AR, KL, COL16A1, GSS*
biological process	response to peptide hormone stimulus	GO:0043434	0.0169	C=399; O=7; E=1.66; R=4.21; rawP=0.0013	*AR, AHSG, FGFR4, CITED1, APOBEC1, KL, PLA2G1B*
biological process	cell-cell signaling	GO:0007267	0.0183	C=1099; O=12; E=4.58; R=2.62; rawP=0.0017	*FGFR4, NMUR2, C1QA, KCNQ3, NPTX1, AR, APOE, STAB1, SYT4, LAMA2, CAV2, GAL3ST4*
biological process	defense response	GO:0006952	0.0185	C=1107; O=12; E=4.62; R=2.60; rawP=0.0018	*AHSG, C1QB, CITED1, APOBEC1, C1QA, C1QC, APOE, IL20, STAB1, KL, IL1RAPL2, NLRC4*
biological process	regulation of intracellular protein kinase cascade	GO:0010627	0.0197	C=683; O=9; E=2.85; R=3.16; rawP=0.0020	*FGFR4, PLA2G1B, GADD45G, APOE, AR, IL20, KL, ROS1, CTH*
biological process	tissue development	GO:0009888	0.0198	C=1449; O=14; E=6.04; R=2.32; rawP=0.0021	*AHSG, PDZRN3, FGFR4, FOXP2, CITED1, AR, PRICKLE1, IL20, CHRDL2, KL, CAV2, ROS1, SOX5, SOX6*
biological process	cellular response to endogenous stimulus	GO:0071495	0.0208	C=564; O=8; E=2.35; R=3.40; rawP=0.0023	*AHSG, FGFR4, APOBEC1, PLA2G1B, KL, COL16A1, SOX6, SOX5*
biological process	response to organic substance	GO:0010033	0.0226	C=1818; O=16; E=7.58; R=2.11; rawP=0.0026	*FGFR4, RERG, KL, GSS, C1QB, AHSG, APOBEC1, CITED1, PLA2G1B, STEAP2, APOE, AR, COL16A1, SOX6, SOX5, CTH*
biological process	regulation of MAPK cascade	GO:0043408	0.0234	C=456; O=7; E=1.90; R=3.68; rawP=0.0028	*FGFR4, PLA2G1B, GADD45G, APOE, AR, KL, ROS1*
biological process	anatomical structure morphogenesis	GO:0009653	0.0283	C=2055; O=17; E=8.57; R=1.98; rawP=0.0035	*FGFR4, PALMD, KCNQ3, GPC4, NPTX1, PRICKLE1, STAB1, LAMA2, FOXP2, TMOD1, AHSG, CITED1, SOSTDC1, AR, APOE, COL9A2, SOX6*
biological process	cellular developmental process	GO:0048869	0.0294	C=2829; O=21; E=11.80; R=1.78; rawP=0.0039	*FGFR4, PALMD, KCNQ3, ASPA, GADD45G, NPTX1, PRICKLE1, LAMA2, ROS1, TMOD1, PDZRN3, CITED1, C1QC, AR, APOE, IL20, CHRDL2, COL9A2, CAV2, SOX5, SOX6*
biological process	cell proliferation	GO:0008283	0.0294	C=1548; O=14; E=6.45; R=2.17; rawP=0.0039	*FGFR4, FOXP2, CITED1, APOBEC1, PLA2G1B, GPC4, RERG, AR, APOE, LIPG, HTR4, ROS1, CAV2, CTH*
biological process	positive regulation of biological process	GO:0048518	0.0299	C=3439; O=24; E=14.34; R=1.67; rawP=0.0041	*FGFR4, ASPA, GADD45G, PRICKLE1, LIPG, LAMA2, HTR4, KL, NLRC4, AHSG, FOXP2, C1QB, CITED1, APOBEC1, C1QA, PLA2G1B, C1QC, AR, APOE, IL20, CAV2, SOX5, SOX6, CTH*
biological process	cell differentiation	GO:0030154	0.0318	C=2665; O=20; E=11.11; R=1.80; rawP=0.0045	*FGFR4, KCNQ3, ASPA, GADD45G, NPTX1, PRICKLE1, LAMA2, ROS1, TMOD1, PDZRN3, CITED1, C1QC, APOE, AR, IL20, CHRDL2, COL9A2, CAV2, SOX5, SOX6*
biological process	cell growth	GO:0016049	0.0336	C=380; O=6; E=1.58; R=3.79; rawP=0.0049	*AR, AHSG, APOE, ROS1, RERG, CTH*
biological process	skeletal system development	GO:0001501	0.0358	C=387; O=6; E=1.61; R=3.72; rawP=0.0054	*AHSG, PAPSS2, CHRDL2, COL9A2, SOX5, SOX6*

biological process	regulation of response to stimulus	GO:0048583	0.0358	C=2336; O=18; E=9.74; R=1.85; rawP=0.0056	*FGFR4, GADD45G, PRICKLE1, KL, ROS1, NLRC4, C1QB, AHSG, CITED1, C1QA, SOSTDC1, PLA2G1B, C1QC, APOE, AR, IL20, CAV2, CTH*
biological process	response to stimulus	GO:0050896	0.0358	C=6636; O=38; E=27.67; R=1.37; rawP=0.0057	*PAPSS2, RERG, GADD45G, PKIG, LIPG, STAB1, GSS, FOXP2, C1QB, NMUR2, APOBEC1, C1QC, STEAP2, APOE, CAV2, COL9A2, SOX6, CTH, FGFR4, KCNQ3, PRICKLE1, LAMA2, KL, HTR4, ROS1, NLRC4, AHSG, CITED1, C1QA, SOSTDC1, PLA2G1B, AR, IL20, IL1RAPL2, COL16A1, LPAR4, SOX5, CYBRD1*
biological process	MAPK cascade	GO:0000165	0.0369	C=526; O=7; E=2.19; R=3.19; rawP=0.0061	*FGFR4, PLA2G1B, GADD45G, APOE, AR, KL, ROS1*
biological process	transport	GO:0006810	0.0369	C=3338; O=23; E=13.92; R=1.65; rawP=0.0062	*LCA5, KCNQ3, NPTX1, PRICKLE1, PKIG, LIPG, STAB1, ROS1, NLRC4, AHSG, NMUR2, APOBEC1, CITED1, PLA2G1B, SLC16A2, ABCG8, STEAP2, AR, APOE, SLC6A15, SYT4, CAV2, CYBRD1*
biological process	cell development	GO:0048468	0.0371	C=1461; O=13; E=6.09; R=2.13; rawP=0.0064	*PDZRN3, TMOD1, CITED1, KCNQ3, ASPA, NPTX1, APOE, LAMA2, CAV2, COL9A2, ROS1, SOX5, SOX6*
biological process	single organism signaling	GO:0044700	0.0391	C=4646; O=29; E=19.37; R=1.50; rawP=0.0071	*FGFR4, KCNQ3, ASPA, RERG, GADD45G, NPTX1, PRICKLE1, PKIG, STAB1, LAMA2, HTR4, KL, ROS1, NLRC4, GAL3ST4, AHSG, CITED1, NMUR2, SOSTDC1, C1QA, PLA2G1B, AR, APOE, IL20, SYT4, COL16A1, CAV2, LPAR4, CTH*
cellular component	extracellular matrix part	GO:0044420	0.0016	C=185; O=6; E=0.71; R=8.51; rawP=7.30e-05	*C1QB, C1QA, C1QC, LAMA2, COL9A2, COL16A1*
cellular component	extracellular region part	GO:0044421	0.0016	C=1099; O=14; E=4.19; R=3.34; rawP=5.40e-05	*AHSG, C1QB, SOSTDC1, C1QA, PLA2G1B, GPC4, C1QC, APOE, IL20, LIPG, LAMA2, KL, COL16A1, COL9A2*
cellular component	extracellular matrix	GO:0031012	0.0029	C=426; O=8; E=1.62; R=4.93; rawP=0.0002	*AHSG, C1QB, C1QA, GPC4, C1QC, LAMA2, COL9A2, COL16A1*
cellular component	proteinaceous extracellular matrix	GO:0005578	0.0043	C=360; O=7; E=1.37; R=5.10; rawP=0.0004	*C1QB, LAMA2, C1QA, COL16A1, GPC4, COL9A2, C1QC*
cellular component	extracellular space	GO:0005615	0.012	C=856; O=10; E=3.26; R=3.06; rawP=0.0014	*AHSG, C1QB, SOSTDC1, C1QA, PLA2G1B, GPC4, APOE, IL20, LIPG, KL*
cellular component	extracellular region	GO:0005576	0.0401	C=2140; O=16; E=8.16; R=1.96; rawP=0.0056	*FGFR4, GPC4, LIPG, LAMA2, KL, C1QB, AHSG, C1QA, SOSTDC1, PLA2G1B, C1QC, APOE, IL20, CHRDL2, COL9A2, COL16A1*
molecular function	protein dimerization activity	GO:0046983	0.0078	C=972; O=12; E=3.69; R=3.25; rawP=0.0002	*C1QB, FOXP2, CITED1, ANKS6, ABCG8, AR, APOE, CAV2, SOX5, SOX6, GSS, NLRC4*
molecular function	protein homodimerization activity	GO:0042803	0.0254	C=571; O=8; E=2.17; R=3.69; rawP=0.0013	*C1QB, FOXP2, CITED1, ANKS6, APOE, CAV2, GSS, NLRC4*

D10 *vs.* D150. Upregulated D150					
**Database**	**Name**	**ID**	**adjP**	**Statistics**	**Genes**
biological process	extracellular structure organization	GO:0043062	0.000000733	C=212; O=12; E=1.28; R=9.41; rawP=4.90e-09	*APLP1, POSTN, COL5A2, HSPG2, COL4A2, LEPREL2, COL1A1, COL18A1, COL4A1, TNFRSF11B, GAS6, COL21A1*
biological process	extracellular matrix organization	GO:0030198	0.000000733	C=211; O=12; E=1.27; R=9.45; rawP=4.65e-09	*APLP1, POSTN, COL5A2, HSPG2, COL4A2, LEPREL2, COL1A1, COL18A1, COL4A1, TNFRSF11B, GAS6, COL21A1*
biological process	organ morphogenesis	GO:0009887	0.0000218	C=802; O=19; E=4.82; R=3.94; rawP=2.19e-07	*ODAM, DCN, COL5A2, HSPG2, COL1A1, HOXC9, COL18A1, CDX2, TNC, ALX3, APLP1, GATA6, ALPL, HOXA5, DIXDC1, TNFRSF11B, CXCL12, ESR1, LY6H*
biological process	locomotion	GO:0040011	0.0000588	C=1245; O=23; E=7.49; R=3.07; rawP=7.87e-07	*COL5A2, COL4A2, PTPRU, ACVR1B, COL1A1, RET, SELL, COL18A1, RELN, GAS6, ABLIM2, CSF3R, SPP1, CCL26, HOXA5, SPON2, DAB2IP, COL4A1, DIXDC1, CXCL12, UNC5A, SEMA3A, TLX3*
biological process	anatomical structure morphogenesis	GO:0009653	0.0000957	C=2055; O=30; E=12.36; R=2.43; rawP=1.60e-06	*ODAM, DCN, COL5A2, HSPG2, COL4A2, COL1A1, RET, HOXC9, COL18A1, RELN, CDX2, TNC, SHROOM4, ABLIM2, ALX3, SPP1, APLP1, GATA6, HOXA5, ALPL, SPON2, TNFRSF11B, COL4A1, DAB2IP, DIXDC1, CXCL12, UNC5A, SEMA3A, ESR1, LY6H*
biological process	system development	GO:0048731	0.0002	C=3521; O=41; E=21.18; R=1.94; rawP=3.18e-06	*ODAM, DCN, ADAM19, HSPG2, COL4A2, RET, COL18A1, GAS6, CDX2, TNC, SNTA1, INSC, SPP1, APLP1, GJB1, SPARC, ALPL, SPON2, COL4A1, DAB2IP, DIXDC1, CXCL12, COL5A2, COL1A1, ACVR1B, GJB2, HOXC9, RELN, SHROOM4, ALX3, ABLIM2, POSTN, GATA6, HOXA5, CPLX2, TNFRSF11B, UNC5A, SEMA3A, ESR1, TLX3, LY6H*
biological process	organ development	GO:0048513	0.0002	C=2552; O=33; E=15.35; R=2.15; rawP=5.84e-06	*ODAM, DCN, ADAM19, HSPG2, RET, COL18A1, GAS6, CDX2, TNC, SNTA1, INSC, SPP1, APLP1, SPARC, ALPL, COL4A1, DAB2IP, DIXDC1, CXCL12, COL5A2, COL1A1, ACVR1B, GJB2, HOXC9, RELN, ALX3, SHROOM4, GATA6, HOXA5, TNFRSF11B, ESR1, SEMA3A, LY6H*
biological process	multicellular organismal process	GO:0032501	0.0003	C=5644; O=54; E=33.95; R=1.59; rawP=1.21e-05	*ODAM, KEL, DCN, ADAM19, HSPG2, COL4A2, RET, SELL, COL18A1, GAS6, CDX2, TNC, OTOS, SNTA1, INSC, CSF2RB, SPP1, APLP1, GJB1, SPARC, PDE10A, ALPL, SPON2, COL4A1, DAB2IP, DIXDC1, CXCL12, BGN, GRPR, COL5A2, COL1A1, ACVR1B, GJB2, KCNMA1, HOXC9, MGAM, RELN, SHROOM4, ABLIM2, ALX3, GABRB3, POSTN, GATA6, PAH, ADCY8, HOXA5, CPLX2, TNFRSF11B, MAPK13, UNC5A, SEMA3A, ESR1, TLX3, LY6H*
biological process	single-multicellular organism process	GO:0044707	0.0003	C=5612; O=54; E=33.76; R=1.60; rawP=9.94e-06	*ODAM, KEL, DCN, ADAM19, HSPG2, COL4A2, RET, SELL, COL18A1, GAS6, CDX2, TNC, OTOS, SNTA1, INSC, CSF2RB, SPP1, APLP1, GJB1, SPARC, PDE10A, ALPL, SPON2, COL4A1, DAB2IP, DIXDC1, CXCL12, BGN, GRPR, COL5A2, COL1A1, ACVR1B, GJB2, KCNMA1, HOXC9, MGAM, RELN, SHROOM4, ABLIM2, ALX3, GABRB3, POSTN, GATA6, PAH, ADCY8, HOXA5, CPLX2, TNFRSF11B, MAPK13, UNC5A, SEMA3A, ESR1, TLX3, LY6H*
biological process	tissue development	GO:0009888	0.0003	C=1449; O=23; E=8.72; R=2.64; rawP=1.04e-05	*ODAM, DCN, COL5A2, HSPG2, ACVR1B, COL1A1, RET, COL18A1, GAS6, TNC, INSC, SNTA1, SPP1, POSTN, GATA6, HOXA5, ALPL, DAB2IP, COL4A1, DIXDC1, CXCL12, SEMA3A, ESR1*
biological process	axonogenesis	GO:0007409	0.0003	C=526; O=13; E=3.16; R=4.11; rawP=1.47e-05	*SPP1, COL5A2, COL4A2, COL1A1, SPON2, RELN, COL4A1, DIXDC1, CXCL12, UNC5A, SEMA3A, TNC, ABLIM2*
biological process	cell migration	GO:0016477	0.0003	C=841; O=17; E=5.06; R=3.36; rawP=9.00e-06	*PTPRU, ACVR1B, COL1A1, RET, SELL, COL18A1, RELN, GAS6, CSF3R, CCL26, SPP1, HOXA5, DIXDC1, DAB2IP, CXCL12, SEMA3A, TLX3*
biological process	taxis	GO:0042330	0.0003	C=595; O=14; E=3.58; R=3.91; rawP=1.16e-05	*SPP1, CCL26, COL5A2, COL4A2, COL1A1, SPON2, RELN, COL4A1, CXCL12, GAS6, UNC5A, SEMA3A, ABLIM2, CSF3R*
biological process	chemotaxis	GO:0006935	0.0003	C=595; O=14; E=3.58; R=3.91; rawP=1.16e-05	*SPP1, CCL26, COL5A2, COL4A2, COL1A1, SPON2, RELN, COL4A1, CXCL12, GAS6, UNC5A, SEMA3A, ABLIM2*
biological process	cell morphogenesis involved in differentiation	GO:0000904	0.0004	C=704; O=15; E=4.23; R=3.54; rawP=1.77e-05	*SPP1, COL5A2, COL4A2, COL1A1, SPON2, COL18A1, RELN, COL4A1, DAB2IP, DIXDC1, CXCL12, UNC5A, SEMA3A, TNC, ABLIM2*
biological process	localization of cell	GO:0051674	0.0004	C=910; O=17; E=5.47; R=3.11; rawP=2.50e-05	*PTPRU, ACVR1B, COL1A1, RET, SELL, COL18A1, RELN, GAS6, CSF3R, CCL26, SPP1, HOXA5, DIXDC1, DAB2IP, CXCL12, SEMA3A, TLX3*
biological process	cell motility	GO:0048870	0.0004	C=910; O=17; E=5.47; R=3.11; rawP=2.50e-05	*PTPRU, ACVR1B, COL1A1, RET, SELL, COL18A1, RELN, GAS6, CSF3R, CCL26, SPP1, HOXA5, DIXDC1, DAB2IP, CXCL12, SEMA3A, TLX3*
biological process	response to external stimulus	GO:0009605	0.0005	C=1323; O=21; E=7.96; R=2.64; rawP=2.79e-05	*DCN, COL5A2, COL4A2, COL1A1, RET, RELN, GAS6, ABLIM2, CSF3R, CCL26, SPP1, SPARC, SPON2, ALPL, COL4A1, TNFRSF11B, MAPK13, CXCL12, UNC5A, SEMA3A, NT5E*
biological process	anatomical structure development	GO:0048856	0.0006	C=4030; O=42; E=24.24; R=1.73; rawP=4.18e-05	*ODAM, DCN, ADAM19, HSPG2, COL4A2, RET, COL18A1, GAS6, CDX2, TNC, SNTA1, INSC, SPP1, APLP1, GJB1, SPARC, ALPL, SPON2, COL4A1, DAB2IP, DIXDC1, CXCL12, COL5A2, COL1A1, ACVR1B, GJB2, HOXC9, RELN, SHROOM4, ALX3, ABLIM2, POSTN, GATA6, HOXA5, CPLX2, MMP25, TNFRSF11B, UNC5A, SEMA3A, ESR1, TLX3, LY6H*
biological process	cell morphogenesis involved in neuron differentiation	GO:0048667	0.0006	C=575; O=13; E=3.46; R=3.76; rawP=3.74e-05	*SPP1, COL5A2, COL4A2, COL1A1, SPON2, RELN, COL4A1, DIXDC1, CXCL12, UNC5A, SEMA3A, TNC, ABLIM2*
biological process	neuron projection morphogenesis	GO:0048812	0.0006	C=583; O=13; E=3.51; R=3.71; rawP=4.31e-05	*SPP1, COL5A2, COL4A2, COL1A1, SPON2, RELN, COL4A1, DIXDC1, CXCL12, UNC5A, SEMA3A, TNC, ABLIM2*
biological process	nervous system development	GO:0007399	0.0007	C=1724; O=24; E=10.37; R=2.31; rawP=5.60e-05	*COL5A2, HSPG2, COL4A2, ACVR1B, COL1A1, RET, RELN, GAS6, TNC, SHROOM4, ABLIM2, INSC, APLP1, SPP1, GJB1, SPON2, CPLX2, DIXDC1, COL4A1, CXCL12, UNC5A, SEMA3A, TLX3, LY6H*
biological process	axon guidance	GO:0007411	0.0007	C=359; O=10; E=2.16; R=4.63; rawP=5.76e-05	*COL5A2, COL4A2, COL1A1, SPON2, COL4A1, RELN, CXCL12, UNC5A, SEMA3A, ABLIM2*
biological process	neuron projection development	GO:0031175	0.0009	C=704; O=14; E=4.23; R=3.31; rawP=7.38e-05	*SPP1, COL5A2, COL4A2, COL1A1, SPON2, RET, RELN, COL4A1, DIXDC1, CXCL12, UNC5A, SEMA3A, TNC, ABLIM2*
biological process	multicellular organismal development	GO:0007275	0.001	C=4077; O=41; E=24.52; R=1.67; rawP=0.0001	*ODAM, DCN, ADAM19, HSPG2, COL4A2, RET, COL18A1, GAS6, CDX2, TNC, SNTA1, INSC, SPP1, APLP1, GJB1, SPARC, ALPL, SPON2, COL4A1, DAB2IP, DIXDC1, CXCL12, COL5A2, COL1A1, ACVR1B, GJB2, HOXC9, RELN, SHROOM4, ALX3, ABLIM2, POSTN, GATA6, HOXA5, CPLX2, TNFRSF11B, UNC5A, SEMA3A, ESR1, TLX3, LY6H*
biological process	cell development	GO:0048468	0.001	C=1461; O=21; E=8.79; R=2.39; rawP=0.0001	*COL5A2, COL4A2, COL1A1, RET, COL18A1, RELN, CDX2, TNC, ABLIM2, SNTA1, SPP1, HOXA5, SPON2, DAB2IP, COL4A1, DIXDC1, CXCL12, UNC5A, SEMA3A, ESR1, TLX3*
biological process	cell morphogenesis	GO:0000902	0.001	C=945; O=16; E=5.68; R=2.81; rawP=0.0001	*SPP1, COL5A2, COL4A2, COL1A1, SPON2, COL18A1, RELN, COL4A1, DAB2IP, DIXDC1, CXCL12, UNC5A, SEMA3A, TNC, SHROOM4, ABLIM2*
biological process	regulation of cell morphogenesis involved in differentiation	GO:0010769	0.001	C=187; O=7; E=1.12; R=6.22; rawP=0.0001	*RELN, SPP1, DIXDC1, DAB2IP, CXCL12, SEMA3A, COL1A1*
biological process	regulation of cell migration	GO:0030334	0.001	C=396; O=10; E=2.38; R=4.20; rawP=0.0001	*CCL26, PTPRU, ACVR1B, COL1A1, RET, COL18A1, DAB2IP, GAS6, CXCL12, SEMA3A*
biological process	regulation of anatomical structure morphogenesis	GO:0022603	0.0018	C=591; O=12; E=3.55; R=3.38; rawP=0.0002	*SPP1, GATA6, COL4A2, COL1A1, HOXA5, DAB2IP, DIXDC1, TNFRSF11B, RELN, CXCL12, SEMA3A, ESR1*
biological process	cell part morphogenesis	GO:0032990	0.0018	C=691; O=13; E=4.16; R=3.13; rawP=0.0002	*SPP1, COL5A2, COL4A2, COL1A1, SPON2, RELN, COL4A1, DIXDC1, CXCL12, UNC5A, SEMA3A, TNC, ABLIM2*
biological process	cell projection morphogenesis	GO:0048858	0.0018	C=679; O=13; E=4.08; R=3.18; rawP=0.0002	*SPP1, COL5A2, COL4A2, COL1A1, SPON2, RELN, COL4A1, DIXDC1, CXCL12, UNC5A, SEMA3A, TNC, ABLIM2*
biological process	regulation of cell motility	GO:2000145	0.0018	C=418; O=10; E=2.51; R=3.98; rawP=0.0002	*CCL26, PTPRU, ACVR1B, COL1A1, RET, COL18A1, DAB2IP, GAS6, CXCL12, SEMA3A*
biological process	response to chemical stimulus	GO:0042221	0.0018	C=2746; O=31; E=16.52; R=1.88; rawP=0.0002	*DCN, COL5A2, COL4A2, COL1A1, PLA2G5, KCNMA1, RET, SELL, COL18A1, RELN, GAS6, CYP4F3, ABLIM2, CSF3R, CSF2RB, SPP1, APLP1, CCL26, IFIT2, GATA6, SPARC, ADCY8, ALPL, SPON2, COL4A1, DAB2IP, TNFRSF11B, CXCL12, UNC5A, SEMA3A, ESR1*
biological process	cellular component morphogenesis	GO:0032989	0.0024	C=1000; O=16; E=6.02; R=2.66; rawP=0.0003	*SPP1, COL5A2, COL4A2, COL1A1, SPON2, COL18A1, RELN, COL4A1, DAB2IP, DIXDC1, CXCL12, UNC5A, SEMA3A, TNC, SHROOM4, ABLIM2*
biological process	neuron development	GO:0048666	0.0024	C=798; O=14; E=4.80; R=2.92; rawP=0.0003	*SPP1, COL5A2, COL4A2, COL1A1, SPON2, RET, RELN, COL4A1, DIXDC1, CXCL12, UNC5A, SEMA3A, TNC, ABLIM2*
biological process	response to endogenous stimulus	GO:0009719	0.0024	C=1004; O=16; E=6.04; R=2.65; rawP=0.0003	*SPP1, APLP1, GATA6, COL5A2, COL4A2, SPARC, ADCY8, COL1A1, PLA2G5, ALPL, SELL, COL4A1, DAB2IP, TNFRSF11B, CXCL12, ESR1*
biological process	developmental process	GO:0032502	0.0028	C=4572; O=43; E=27.50; R=1.56; rawP=0.0004	*ODAM, DCN, ADAM19, HSPG2, COL4A2, RET, COL18A1, GAS6, CDX2, TNC, SNTA1, INSC, SPP1, APLP1, GJB1, SPARC, ALPL, SPON2, COL4A1, DAB2IP, DIXDC1, CXCL12, COL5A2, PTPRU, COL1A1, ACVR1B, GJB2, HOXC9, RELN, SHROOM4, ABLIM2, ALX3, POSTN, GATA6, HOXA5, CPLX2, MMP25, TNFRSF11B, UNC5A, SEMA3A, ESR1, TLX3, LY6H*
biological process	anatomical structure formation involved in morphogenesis	GO:0048646	0.0028	C=1594; O=21; E=9.59; R=2.19; rawP=0.0004	*COL5A2, HSPG2, COL4A2, COL1A1, RET, COL18A1, RELN, CDX2, TNC, ABLIM2, SPP1, GATA6, HOXA5, SPON2, ALPL, DIXDC1, COL4A1, CXCL12, UNC5A, SEMA3A, ESR1*
biological process	regulation of locomotion	GO:0040012	0.0028	C=449; O=10; E=2.70; R=3.70; rawP=0.0004	*CCL26, PTPRU, ACVR1B, COL1A1, RET, COL18A1, DAB2IP, GAS6, CXCL12, SEMA3A*

celullar component	extracellular matrix	GO:0031012	0.000000000134	C=426; O=19; E=2.50; R=7.61; rawP=4.97e-12	*ODAM, DCN, COL5A2, HSPG2, COL4A2, COL1A1, COL18A1, RELN, COL21A1, TNC, APLP1, POSTN, SPARC, SPON2, CPXM2, MMP25, COL4A1, TNFRSF11B, BGN*
celullar component	proteinaceous extracellular matrix	GO:0005578	0.000000000134	C=360; O=18; E=2.11; R=8.53; rawP=2.88e-12	*ODAM, DCN, COL5A2, HSPG2, COL4A2, COL1A1, COL18A1, RELN, COL21A1, TNC, APLP1, POSTN, SPARC, SPON2, MMP25, COL4A1, TNFRSF11B, BGN*
celullar component	extracellular matrix part	GO:0044420	0.0000000153	C=185; O=12; E=1.08; R=11.06; rawP=8.50e-10	*ODAM, DCN, APLP1, COL5A2, HSPG2, COL4A2, SPARC, COL1A1, COL18A1, COL4A1, COL21A1, TNC*
celullar component	extracellular region	GO:0005576	0.0000000806	C=2140; O=35; E=12.55; R=2.79; rawP=5.97e-09	*ODAM, DCN, HSPG2, COL4A2, PRSS35, COL18A1, GAS6, PIGR, OTOS, TNC, CSF3R, APLP1, SPP1, CCL26, SPARC, ALPL, SPON2, CPXM2, COL4A1, CXCL12, BGN, MUC15, RNASE1, PLA2G2C, COL5A2, COL1A1, PLA2G5, RELN, COL21A1, GP2, POSTN, NXPH4, MMP25, TNFRSF11B, SEMA3A*
celullar component	extracellular region part	GO:0044421	0.000000157	C=1099; O=24; E=6.44; R=3.72; rawP=1.45e-08	*ODAM, DCN, COL5A2, HSPG2, COL4A2, COL1A1, COL18A1, RELN, GAS6, COL21A1, TNC, APLP1, CCL26, SPP1, POSTN, SPARC, ALPL, SPON2, CPXM2, MMP25, COL4A1, TNFRSF11B, CXCL12, BGN*
celullar component	basement membrane	GO:0005604	0.00000692	C=89; O=7; E=0.52; R=13.41; rawP=8.97e-07	*COL18A1, APLP1, COL4A1, HSPG2, COL4A2, SPARC, TNC*
celullar component	collagen	GO:0005581	0.00000692	C=89; O=7; E=0.52; R=13.41; rawP=8.97e-07	*COL18A1, DCN, COL4A1, COL5A2, COL4A2, COL21A1*
celullar component	endoplasmic reticulum lumen	GO:0005788	0.0000477	C=170; O=8; E=1.00; R=8.02; rawP=7.06e-06	*COL5A2, COL4A2, LEPREL2, COL1A1, COL18A1, COL4A1, GAS6, COL21A1*
celullar component	extracellular space	GO:0005615	0.003	C=856; O=14; E=5.02; R=2.79; rawP=0.0005	*DCN, SPP1, CCL26, HSPG2, SPARC, COL1A1, ALPL, CPXM2, COL18A1, RELN, TNFRSF11B, CXCL12, GAS6, TNC*
celullar component	endoplasmic reticulum part	GO:0044432	0.0146	C=919; O=13; E=5.39; R=2.41; rawP=0.0027	*RETSAT, FADS2, GJB1, COL5A2, COL4A2, LEPREL2, COL1A1, COL18A1, COL4A1, GAS6, MBOAT2, COL21A1, CYP4F3*
celullar component	endoplasmic reticulum	GO:0005783	0.0309	C=1282; O=15; E=7.52; R=2.00; rawP=0.0076	*RETSAT, FADS2, IFIT2, GJB1, COL5A2, COL4A2, LEPREL2, COL1A1, PLCB4, COL18A1, COL4A1, GAS6, MBOAT2, COL21A1, CYP4F3*
celullar component	apical part of cell	GO:0045177	0.0309	C=294; O=6; E=1.72; R=3.48; rawP=0.0077	*INSC, MGAM, SPP1, GP2, SHROOM4, KCNMA1*
celullar component	cell periphery	GO:0071944	0.0309	C=4377; O=37; E=25.67; R=1.44; rawP=0.0080	*KEL, GPR33, GPRC5C, HSPG2, RET, SELL, PIGR, CSF3R, CSF2RB, SNTA1, APLP1, GJB1, ALPL, DIXDC1, DAB2IP, CXCL12, BGN, MUC15, NT5E, FADS2, GRPR, PTPRU, ACVR1B, GJB2, KCNMA1, PLA2G5, SLC2A10, MGAM, GP2, SHROOM4, GABRB3, ADCY8, MMP25, UNC5A, ESR1, LY6H, LPHN3*
celullar component	plasma membrane part	GO:0044459	0.0309	C=1918; O=20; E=11.25; R=1.78; rawP=0.0073	*FADS2, GPRC5C, GRPR, PTPRU, GJB2, ACVR1B, KCNMA1, RET, SELL, MGAM, PIGR, GP2, SHROOM4, CSF3R, CSF2RB, GJB1, GABRB3, DAB2IP, CXCL12, ESR*
celullar component	plasma membrane	GO:0005886	0.0367	C=4289; O=36; E=25.15; R=1.43; rawP=0.0102	*KEL, GPR33, GPRC5C, HSPG2, RET, SELL, PIGR, CSF3R, CSF2RB, SNTA1, APLP1, GJB1, ALPL, DAB2IP, CXCL12, BGN, MUC15, NT5E, FADS2, GRPR, PTPRU, ACVR1B, GJB2, KCNMA1, PLA2G5, SLC2A10, MGAM, GP2, SHROOM4, GABRB3, ADCY8, MMP25, UNC5A, ESR1, LY6H, LPHN3*
celullar component	intrinsic to membrane	GO:0031224	0.0422	C=5437; O=43; E=31.88; R=1.35; rawP=0.0125	*C1orf95, KEL, GPR33, ADAM19, CHST1, GPRC5C, KIAA1644, RET, SELL, PIGR, TMEM145, CSF3R, CSF2RB, APLP1, GJB1, ALPL, DAB2IP, MUC15, NT5E, MS4A15, FADS2, GRPR, PTPRU, ACVR1B, GJB2, KCNMA1, SLC2A10, MGAM, WBSCR17, GP2, HEPACAM2, CYP4F3, KCNE3, GABRB3, ADCY8, ABHD3, SERTM1, MMP25, UNC5A, ESR1, MBOAT2, LY6H, LPHN3*
molecular function	carbohydrate derivative binding	GO:0097367	0.02	C=189; O=6; E=1.10; R=5.45; rawP=0.0008	*SELL, DCN, APLP1, BGN, POSTN, PLA2G5*
molecular function	glycosaminoglycan binding	GO:0005539	0.02	C=174; O=6; E=1.01; R=5.92; rawP=0.0005	*SELL, DCN, APLP1, BGN, POSTN, PLA2G5*

**D10 *vs.* D120. Upregulated D10**					
**Database**	**Name**	**ID**	**adjP**	**Statistics**	**Genes**
biological process	cell cycle	GO:0007049	0.0282	C=1393; O=12; E=4.00; R=3.00; rawP=0.0004	*HELLS, KIAA0101, HJURP, KLHL13, GADD45G, FANCD2, MAPK4, CENPE, PDPN, IQGAP3, CETN1, CENPP*
biological process	developmental process	GO:0032502	0.0423	C=4572; O=23; E=13.13; R=1.75; rawP=0.0013	*PALMD, CECR1, ASPA, PAPSS2, TREM2, GADD45G, PAX2, STAB1, LAMA2, KL, ROBO3, C1QB, PLA2R1, HELLS, NMUR2, C1QC, APOE, SERPINI1, CENPE, PDPN, IQGAP3, CHRDL2, IL1RAPL2*
biological process	multicellular organismal development	GO:0007275	0.0423	C=4077; O=21; E=11.70; R=1.79; rawP=0.0019	*CECR1, ASPA, PAPSS2, TREM2, GADD45G, PAX2, STAB1, LAMA2, KL, ROBO3, C1QB, HELLS, NMUR2, C1QC, APOE, SERPINI1, CENPE, PDPN, IQGAP3, CHRDL2, IL1RAPL2*
biological process	system development	GO:0048731	0.0423	C=3521; O=19; E=10.11; R=1.88; rawP=0.0021	*ASPA, PAPSS2, TREM2, PAX2, STAB1, LAMA2, KL, ROBO3, C1QB, HELLS, NMUR2, C1QC, APOE, SERPINI1, CENPE, PDPN, IQGAP3, CHRDL2, IL1RAPL2*
biological process	response to stimulus	GO:0050896	0.0423	C=6636; O=29; E=19.05; R=1.52; rawP=0.0016	*TFPI2, LTBP1, PAPSS2, TREM2, GADD45G, PAX2, FANCD2, RGS20, MAPK4, STAB1, LAMA2, KL, HTR4, ROBO3, NLRC4, CETN1, C1QB, PLA2R1, KIAA0101, NMUR2, APOBEC1, C1QA, C1QC, STEAP2, APOE, CENPE, PDPN, IQGAP3, IL1RAPL2*
biological process	response to stress	GO:0006950	0.0083	C=2952; O=20; E=8.47; R=2.36; rawP=5.92e-05	*TFPI2, PAPSS2, GADD45G, PAX2, FANCD2, STAB1, KL, NLRC4, CETN1, C1QB, PLA2R1, KIAA0101, NMUR2, APOBEC1, C1QA, C1QC, APOE, CENPE, PDPN, IL1RAPL2*
biological process	defense response	GO:0006952	0.0423	C=1107; O=10; E=3.18; R=3.15; rawP=0.0009	*C1QB, APOBEC1, C1QA, C1QC, APOE, STAB1, PDPN, KL, IL1RAPL2, NLRC4*
cellular component	extracellular region	GO:0005576	0.0488	C=2140; O=13; E=6.15; R=2.11; rawP=0.0061	*C1QB, CECR1, TFPI2, PLA2R1, LTBP1, C1QA, TREM2, C1QC, APOE, SERPINI1, LAMA2, CHRDL2, KL*
cellular component	extracellular matrix	GO:0031012	0.0208	C=426; O=6; E=1.22; R=4.90; rawP=0.0013	*C1QB, TFPI2, LTBP1, C1QA, C1QC, LAMA2*
cellular component	extracellular region part	GO:0044421	0.0395	C=1099; O=9; E=3.16; R=2.85; rawP=0.0037	*APOE, C1QB, CECR1, TFPI2, LTBP1, LAMA2, C1QA, KL, C1QC*
cellular component	proteinaceous extracellular matrix	GO:0005578	0.0192	C=360; O=6; E=1.03; R=5.80; rawP=0.0006	*C1QB, TFPI2, LTBP1, C1QA, C1QC, LAMA2*

D10 *vs.* D120. Upregulated D120					
**Database**	**Name**	**ID**	**adjP**	**Statistics**	**Genes**
biological process	locomotion	GO:0040011	0.0060	C=1245; O=15; E=5.28; R=2.84; rawP=0.0002	*CCL26, TTN, ADORA1, COL4A2, PTPRU, ITGA7, TRPC6, RET, RELN, COL4A1, DAB2IP, GAS6, UNC5A, TLX3, ABLIM2*
biological process	multicellular organismal process	GO:0032501	0.0008	C=5644; O=41; E=23.92; R=1.71; rawP=1.00e-05	*KEL, TTN, COL4A2, ETV5, RET, GAS6, OTOS, SNTA1, APLP1, GJB1, ADORA1, PDE10A, ALPL, COL4A1, DAB2IP, SMAD6, ZNF750, GRPR, GJB2, MGAM, HOXC9, RELN, ALX3, ABLIM2, ESRRB, GABRB3, PDLIM3, GATA6, PAH, ITGA7, TRPC6, DGKI, CPLX2, GFI1B, MAPK13, TNFRSF11B, UNC5A, TLX3, CHI3L1, LY6H, ABCG1*
biological process	single-organism process	GO:0044699	0.0096	C=7682; O=46; E=32.56; R=1.41; rawP=0.0004	*KEL, TTN, COL4A2, ETV5, RET, GAS6, OTOS, SNTA1, APLP1, CCL26, GJB1, ADORA1, PDE10A, ALPL, COL4A1, DAB2IP, SMAD6, ZNF750, GRPR, PTPRU, GJB2, HOMER2, MGAM, HOXC9, RELN, ALX3, ABLIM2, ESRRB, GABRB3, PDLIM3, GATA6, PAH, ITGA7, DGKI, TRPC6, CPLX2, GFI1B, NXPH4, MAPK13, TNFRSF11B, UNC5A, TLX3, CHI3L1, LY6H, EMR3, ABCG1*
biological process	developmental process	GO:0032502	0.0080	C=4572; O=33; E=19.38; R=1.70; rawP=0.0003	*TTN, COL4A2, ETV5, RET, GAS6, SNTA1, APLP1, GJB1, ADORA1, ALPL, COL4A1, DAB2IP, SMAD6, ZNF750, PTPRU, GJB2, HOXC9, RELN, ALX3, ABLIM2, ESRRB, PDLIM3, GATA6, ITGA7, TRPC6, CPLX2, GFI1B, TNFRSF11B, UNC5A, TLX3, LY6H, CHI3L1, ABCG1*
biological process	single-multicellular organism process	GO:0044707	0.0008	C=5612; O=41; E=23.78; R=1.72; rawP=8.49e-06	*KEL, TTN, COL4A2, ETV5, RET, GAS6, OTOS, SNTA1, APLP1, GJB1, ADORA1, PDE10A, ALPL, COL4A1, DAB2IP, SMAD6, ZNF750, GRPR, GJB2, MGAM, HOXC9, RELN, ALX3, ABLIM2, ESRRB, GABRB3, PDLIM3, GATA6, PAH, ITGA7, TRPC6, DGKI, CPLX2, GFI1B, MAPK13, TNFRSF11B, UNC5A, TLX3, CHI3L1, LY6H, ABCG1*
biological process	regulation of developmental process	GO:0050793	0.0426	C=1436; O=14; E=6.09; R=2.30; rawP=0.0023	*ESRRB, GATA6, COL4A2, ITGA7, ETV5, RET, GFI1B, TNFRSF11B, DAB2IP, RELN, GAS6, TLX3, CHI3L1, ABCG1*
biological process	anatomical structure development	GO:0048856	0.0048	C=4030; O=31; E=17.08; R=1.82; rawP=0.0001	*TTN, COL4A2, GJB2, ETV5, RET, HOXC9, RELN, GAS6, ABLIM2, ALX3, ESRRB, SNTA1, APLP1, GJB1, GATA6, PDLIM3, ADORA1, ITGA7, TRPC6, ALPL, CPLX2, GFI1B, TNFRSF11B, COL4A1, DAB2IP, UNC5A, TLX3, SMAD6, CHI3L1, LY6H, ZNF750*
biological process	multicellular organismal development	GO:0007275	0.0060	C=4077; O=31; E=17.28; R=1.79; rawP=0.0002	*TTN, COL4A2, GJB2, ETV5, RET, HOXC9, RELN, GAS6, ABLIM2, ALX3, ESRRB, SNTA1, APLP1, GJB1, GATA6, PDLIM3, ADORA1, ITGA7, TRPC6, ALPL, CPLX2, GFI1B, TNFRSF11B, COL4A1, DAB2IP, UNC5A, TLX3, SMAD6, CHI3L1, LY6H, ZNF750*
biological process	anatomical structure morphogenesis	GO:0009653	0.0060	C=2055; O=20; E=8.71; R=2.30; rawP=0.0002	*TTN, COL4A2, ETV5, RET, HOXC9, RELN, ABLIM2, ALX3, ESRRB, APLP1, GATA6, ITGA7, ALPL, TRPC6, TNFRSF11B, DAB2IP, COL4A1, UNC5A, CHI3L1, LY6H*
biological process	system development	GO:0048731	0.0008	C=3521; O=31; E=14.92; R=2.08; rawP=8.30e-06	*TTN, COL4A2, GJB2, ETV5, RET, HOXC9, RELN, GAS6, ABLIM2, ALX3, ESRRB, SNTA1, APLP1, GJB1, GATA6, PDLIM3, ADORA1, ITGA7, TRPC6, ALPL, CPLX2, GFI1B, TNFRSF11B, COL4A1, DAB2IP, UNC5A, TLX3, SMAD6, CHI3L1, LY6H, ZNF750*
biological process	organ development	GO:0048513	0.0110	C=2552; O=22; E=10.82; R=2.03; rawP=0.0005	*TTN, GJB2, ETV5, RET, HOXC9, RELN, GAS6, ALX3, SNTA1, ESRRB, APLP1, PDLIM3, GATA6, ITGA7, ALPL, GFI1B, TNFRSF11B, COL4A1, DAB2IP, CHI3L1, LY6H, ZNF750*
biological process	response to external stimulus	GO:0009605	0.0048	C=1323; O=16; E=5.61; R=2.85; rawP=9.07e-05	*TTN, COL4A2, RET, RELN, GAS6, ABLIM2, CCL26, ADORA1, ALPL, TRPC6, COL4A1, TNFRSF11B, MAPK13, UNC5A, CHI3L1, NT5E*
biological process	response to wounding	GO:0009611	0.0422	C=1109; O=12; E=4.70; R=2.55; rawP=0.0021	*CCL26, TTN, CHST1, GATA6, ADORA1, PDE10A, DGKI, TRPC6, MAPK13, GAS6, CHI3L1, NT5E*
cellular component	extracellular region	GO:0005576	0.0270	C=2140; O=18; E=8.41; R=2.14; rawP=0.0012	*TTN, RNASE1, COL4A2, PRSS35, SLPI, RELN, GAS6, GP2, OTOS, CCL26, APLP1, ALPL, CPXM2, NXPH4, TNFRSF11B, COL4A1, CHI3L1, EMR3*
cellular component	extracellular matrix	GO:0031012	0.0135	C=426; O=8; E=1.67; R=4.78; rawP=0.0003	*APLP1, COL4A2, SLPI, CPXM2, COL4A1, TNFRSF11B, RELN, CHI3L1*
cellular component	extracellular region part	GO:0044421	0.0394	C=1099; O=11; E=4.32; R=2.55; rawP=0.0035	*APLP1, CCL26, COL4A2, ALPL, CPXM2, RELN, COL4A1, TNFRSF11B, GAS6, CHI3L1, EMR3*
cellular component	proteinaceous extracellular matrix	GO:0005578	0.0394	C=360; O=6; E=1.41; R=4.24; rawP=0.0029	*RELN, APLP1, TNFRSF11B, COL4A1, COL4A2, CHI3L1*

D50 *vs.* D150. Upregulated D50					
**Database**	**Name**	**ID**	**adjP**	**Statistics**	**Genes**
biological process	multicellular organismal process	GO:0032501	0.0274	C=5644; O=10; E=5.02; R=1.99; rawP=0.0057	*AHSG, EGR1, PLK2, NPTX1, GADD45G, NEFH, EFNA2, IL1RAPL2, COL9A2, DUSP5*
biological process	developmental process	GO:0032502	0.0274	C=4572; O=9; E=4.06; R=2.22; rawP=0.0055	*AHSG, EGR1, NPTX1, GADD45G, NEFH, EFNA2, IL1RAPL2, DUSP5, COL9A2*
biological process	single-multicellular organism process	GO:0044707	0.0274	C=5612; O=10; E=4.99; R=2.01; rawP=0.0055	*AHSG, EGR1, PLK2, NPTX1, GADD45G, NEFH, EFNA2, IL1RAPL2, COL9A2, DUSP5*
biological process	anatomical structure development	GO:0048856	0.0424	C=4030; O=8; E=3.58; R=2.23; rawP=0.0106	*AHSG, EGR1, NPTX1, NEFH, EFNA2, DUSP5, COL9A2, IL1RAPL2*
biological process	multicellular organismal development	GO:0007275	0.0274	C=4077; O=9; E=3.62; R=2.48; rawP=0.0023	*AHSG, EGR1, NPTX1, GADD45G, NEFH, EFNA2, IL1RAPL2, DUSP5, COL9A2*
biological process	nervous system development	GO:0007399	0.0274	C=1724; O=6; E=1.53; R=3.92; rawP=0.0022	*AHSG, EFNA2, IL1RAPL2, COL9A2, NPTX1, NEFH*

Table S3. Gene Ontology (GO) assignment of the DEGs detected between breeds (Churra and Assaf).

Churra *vs.* Assaf. Upregulated Churra					
**Database**	**Name**	**ID**	**adjP**	**Statistics**	**Genes**
biological process	biological adhesion	GO:0022610	0.0259	C=956; O=17; E=7.25; R=2.34; rawP=0.0008	*LAMA4, CNTN5, ROBO2, CDSN, MGP, NLGN1, EFS, ABI3BP, ITGA7, MYBPC1, DSG1, PKP1, COL5A3, FBLN7, NCAM1, CTNND2, BMPR1B*
biological process	cell adhesion	GO:0007155	0.0259	C=954; O=17; E=7.24; R=2.35; rawP=0.0008	*LAMA4, CNTN5, ROBO2, CDSN, MGP, NLGN1, EFS, ABI3BP, ITGA7, MYBPC1, DSG1, PKP1, COL5A3, FBLN7, NCAM1, CTNND2, BMPR1B*
biological process	cellular homeostasis	GO:0019725	0.0259	C=717; O=14; E=5.44; R=2.57; rawP=0.0010	*ACSBG1, NMUR2, UGT8, TRPM8, CACNA1E, DRD3, AQP1, HP, NLGN1, KLK6, SH3TC2, ATP6V1B1, SLC4A11, NPY1R*
biological process	cellular chemical homeostasis	GO:0055082	0.0259	C=631; O=13; E=4.79; R=2.72; rawP=0.0010	*ACSBG1, NMUR2, UGT8, TRPM8, CACNA1E, DRD3, HP, NLGN1, KLK6, SH3TC2, ATP6V1B1, SLC4A11, NPY1R*
biological process	ion homeostasis	GO:0050801	0.0281	C=649; O=13; E=4.92; R=2.64; rawP=0.0013	*ACSBG1, NMUR2, UGT8, TRPM8, CACNA1E, DRD3, HP, NLGN1, KLK6, SH3TC2, ATP6V1B1, SLC4A11, NPY1R*
biological process	cellular ion homeostasis	GO:0006873	0.0259	C=586; O=13; E=4.45; R=2.92; rawP=0.0005	*ACSBG1, NMUR2, UGT8, TRPM8, CACNA1E, DRD3, HP, NLGN1, KLK6, SH3TC2, ATP6V1B1, SLC4A11, NPY1R*
biological process	multicellular organismal process	GO:0032501	0.0281	C=5644; O=59; E=42.82; R=1.38; rawP=0.0012	*MYT1L, SLC22A3, UGT8, ROBO2, FGF13, GRB14, CDSN, MGP, PEBP1, LRP2, AQP1, FGG, CDX2, STX1B, PPP1R9A, ATP6V1B1, MEOX2, NPY1R, INSC, EFEMP1, ACSBG1, NMUR2, DRD3, ZNF521, MYBPC1, COL5A3, ABLIM3, NOTCH3, NCAM1, ST8SIA2, PRG3, CPE, SCG3, KY, LAMA4, BNC1, CNTN5, KLK5, TRPM8, CYP7B1, DUOXA1, CACNA1E, NLGN1, KLK6, TSHZ2, PRELP, FMN2, ITGA7, PIWIL1, CD207, DLG2, KLK7, SH3TC2, PKP1, DUOX2, OSR1, SOX5, CTNND2, BMPR1B*
biological process	single-organism process	GO:0044699	0.0472	C=7682; O=73; E=58.28; R=1.25; rawP=0.0031	*SLC22A3, UGT8, FGF13, MGP, PEBP1, AQP1, FGG, EFS, PTPRG, CDX2, STX1B, PPP1R9A, ATP6V1B1, MEOX2, NMUR2, ZNF521, NOTCH3, PRG3, CPE, SCG3, KY, LAMA4, TRPM8, CYP7B1, KLK6, TSHZ2, PRELP, FMN2, ARHGEF26, PLCH1, CTNND2, BMPR1B, MYT1L, PREX2, ROBO2, CDSN, GRB14, LRP2, NPY1R, EFEMP1, INSC, SORCS1, ACSBG1, MLPH, DRD3, MYBPC1, ABLIM3, COL5A3, ST8SIA2, NCAM1, UCHL1, BNC1, KLK5, CNTN5, CACNA1E, DUOXA1, GPR144, NLGN1, EVC2, ITGA7, CHRM5, PIWIL1, RHBDL1, GPR133, CD207, DLG2, KLK7, SH3TC2, PKP1, DUOX2, DISP2, OSR1, SOX5*
biological process	developmental process	GO:0032502	0.0259	C=4572; O=51; E=34.69; R=1.47; rawP=0.0008	*ATP6V1B1, MEOX2, NPY1R, INSC, EFEMP1, ACSBG1, NMUR2, MLPH, DRD3, ZNF521, COL5A3, ABLIM3, NOTCH3, NCAM1, ST8SIA2, CPE, KY, KRTDAP, LAMA4, BNC1, KLK5, CYP7B1, DUOXA1, NLGN1, KLK6, TSHZ2, PRELP, FMN2, ITGA7, PIWIL1, CD207, DLG2, KLK7, SH3TC2, PKP1, DUOX2, OSR1, SOX5, CTNND2, BMPR1B*
biological process	single-multicellular organism process	GO:0044707	0.0259	C=5612; O=59; E=42.58; R=1.39; rawP=0.0010	*CDX2, STX1B, PPP1R9A, ATP6V1B1, MEOX2, NPY1R, INSC, EFEMP1, ACSBG1, NMUR2, DRD3, ZNF521, MYBPC1, COL5A3, ABLIM3, NOTCH3, NCAM1, ST8SIA2, PRG3, CPE, SCG3, KY, LAMA4, BNC1, CNTN5, KLK5, TRPM8, CYP7B1, DUOXA1, CACNA1E, NLGN1, KLK6, TSHZ2, PRELP, FMN2, ITGA7, PIWIL1, CD207, DLG2, KLK7, SH3TC2, PKP1, DUOX2, OSR1, SOX5, CTNND2, BMPR1B*
biological process	anatomical structure development	GO:0048856	0.0370	C=4030; O=45; E=30.58; R=1.47; rawP=0.0020	*MYT1L, UGT8, ROBO2, FGF13, CDSN, MGP, PEBP1, LRP2, AQP1, CDX2, PPP1R9A, ATP6V1B1, MEOX2, NPY1R, INSC, EFEMP1, ACSBG1, NMUR2, DRD3, COL5A3, ABLIM3, NOTCH3, NCAM1, ST8SIA2, CPE, KY, LAMA4, BNC1, KLK5, CYP7B1, DUOXA1, NLGN1, KLK6, PRELP, ITGA7, PIWIL1, CD207, DLG2, KLK7, SH3TC2, DUOX2, OSR1, SOX5, CTNND2, BMPR1B*
biological process	multicellular organismal development	GO:0007275	0.0259	C=4077; O=49; E=30.93; R=1.58; rawP=0.0002	*MYT1L, UGT8, ROBO2, FGF13, CDSN, MGP, PEBP1, LRP2, AQP1, CDX2, PPP1R9A, ATP6V1B1, MEOX2, NPY1R, INSC, EFEMP1, ACSBG1, NMUR2, DRD3, ZNF521, COL5A3, ABLIM3, NOTCH3, NCAM1, ST8SIA2, CPE, KY, LAMA4, BNC1, KLK5, CYP7B1, DUOXA1, NLGN1, KLK6, TSHZ2, PRELP, FMN2, ITGA7, PIWIL1, CD207, DLG2, KLK7, SH3TC2, PKP1, DUOX2, OSR1, SOX5, CTNND2, BMPR1B*
biological process	system process	GO:0003008	0.0359	C=1695; O=24; E=12.86; R=1.87; rawP=0.0018	*CNTN5, SLC22A3, UGT8, ROBO2, TRPM8, FGF13, CYP7B1, CACNA1E, PEBP1, AQP1, NLGN1, KLK6, STX1B, MEOX2, ATP6V1B1, NPY1R, EFEMP1, NMUR2, ACSBG1, DRD3, MYBPC1, DLG2, SH3TC2, CTNND2*
biological process	system development	GO:0048731	0.0259	C=3521; O=43; E=26.71; R=1.61; rawP=0.0004	*MYT1L, UGT8, ROBO2, FGF13, CDSN, MGP, PEBP1, LRP2, AQP1, CDX2, PPP1R9A, ATP6V1B1, MEOX2, NPY1R, INSC, EFEMP1, ACSBG1, NMUR2, DRD3, COL5A3, ABLIM3, NOTCH3, NCAM1, ST8SIA2, CPE, KY, LAMA4, BNC1, KLK5, CYP7B1, DUOXA1, NLGN1, KLK6, PRELP, ITGA7, CD207, DLG2, KLK7, SH3TC2, DUOX2, OSR1, SOX5, BMPR1B*
biological process	neurological system process	GO:0050877	0.0259	C=1237; O=20; E=9.39; R=2.13; rawP=0.0009	*CNTN5, SLC22A3, UGT8, ROBO2, TRPM8, FGF13, CYP7B1, CACNA1E, PEBP1, NLGN1, KLK6, STX1B, ATP6V1B1, NPY1R, EFEMP1, ACSBG1, DRD3, DLG2, SH3TC2, CTNND2*
biological process	nervous system development	GO:0007399	0.0380	C=1724; O=24; E=13.08; R=1.83; rawP=0.0022	*MYT1L, UGT8, ROBO2, FGF13, DUOXA1, PEBP1, LRP2, AQP1, NLGN1, KLK6, PPP1R9A, INSC, NMUR2, ACSBG1, DRD3, DLG2, SH3TC2, DUOX2, NOTCH3, ABLIM3, NCAM1, ST8SIA2, SOX5, BMPR1B*
biological process	anion transport	GO:0006820	0.0437	C=315; O=8; E=2.39; R=3.35; rawP=0.0027	*SLCO2B1, NMUR2, CLCA4, PLA2G4F, DRD3, SLC4A11, ENPP3, AQP1*
molecular function	peptidase activity	GO:0008233	0.0065	C=582; O=13; E=4.46; R=2.92; rawP=0.0005	*ADAMTS5, KLK5, KLK10, RHBDL1, KLK12, KLK7, KLK13, KLK6, CPE, TMPRSS4, KY, UCHL1, ADAMTS16*
molecular function	peptidase activity, acting on L-amino acid peptides	GO:0070011	0.0130	C=560; O=12; E=4.29; R=2.80; rawP=0.0012	*ADAMTS5, KLK5, KLK10, RHBDL1, KLK12, KLK7, KLK13, KLK6, CPE, TMPRSS4, UCHL1, ADAMTS16*
molecular function	serine hydrolase activity	GO:0017171	0.0015	C=179; O=8; E=1.37; R=5.84; rawP=6.97e-05	*KLK5, KLK10, RHBDL1, KLK12, KLK7, KLK13, KLK6, TMPRSS4*
molecular function	endopeptidase activity	GO:0004175	0.0016	C=377; O=11; E=2.89; R=3.81; rawP=0.0001	*ADAMTS5, KLK5, KLK10, RHBDL1, KLK12, KLK7, KLK13, KLK6, TMPRSS4, UCHL1, ADAMTS16*
molecular function	serine-type peptidase activity	GO:0008236	0.0015	C=177; O=8; E=1.35; R=5.90; rawP=6.44e-05	*KLK5, KLK10, RHBDL1, KLK12, KLK7, KLK13, KLK6, TMPRSS4*
molecular function	serine-type endopeptidase activity	GO:0004252	0.0015	C=155; O=8; E=1.19; R=6.74; rawP=2.50e-05	*KLK5, KLK10, RHBDL1, KLK12, KLK7, KLK13, KLK6, TMPRSS4*
molecular function	passive transmembrane transporter activity	GO:0022803	0.0325	C=420; O=9; E=3.22; R=2.80; rawP=0.0050	*NMUR2, TRPM8, CACNA1E, GABRP, AQP1, STX1B, CLCA4, CACNA2D1, SLC4A11*
molecular function	channel activity	GO:0015267	0.0325	C=420; O=9; E=3.22; R=2.80; rawP=0.0050	*NMUR2, TRPM8, CACNA1E, GABRP, AQP1, STX1B, CLCA4, CACNA2D1, SLC4A11*
molecular function	substrate-specific channel activity	GO:0022838	0.0301	C=402; O=9; E=3.08; R=2.92; rawP=0.0037	*NMUR2, TRPM8, CACNA1E, GABRP, AQP1, STX1B, CLCA4, CACNA2D1, SLC4A11*
molecular function	ion channel activity	GO:0005216	0.0297	C=393; O=9; E=3.01; R=2.99; rawP=0.0032	*NMUR2, TRPM8, CACNA1E, GABRP, AQP1, STX1B, CLCA4, CACNA2D1, SLC4A11*
cellular component	cell projection	GO:0042995	0.0112	C=1230; O=20; E=9.30; R=2.15; rawP=0.0009	*ROBO2, FGF13, PEBP1, LRP2, AQP1, NLGN1, LDB3, PPP1R9A, ATP6V1B1, ARHGEF26, NPY1R, ACPP, EVC2, LY6G6D, PLA2G4F, DRD3, DLG2, NCAM1, CTNND2, BMPR1B*

cellular component	cell periphery	GO:0071944	0.0186	C=4377; O=48; E=33.11; R=1.45; rawP=0.0024	*SLC22A3, ROBO2, FGF13, GRB14, LRP2, AQP1, FGG, STX1B, PTPRG, ATP6V1B1, NPY1R, ACPP, LY6G6D, NMUR2, MLPH, GABRP, DRD3, NOTCH3, NCAM1, CPE, UCHL1, ENPP3, SLCO2B1, CNTN5, TRPM8, SLC14A1, CACNA1E, DUOXA1, GPR144, NLGN1, EVC2,*
cellular component	apical part of cell	GO:0045177	0.0017	C=294; O=10; E=2.22; R=4.50; rawP=8.04e-05	*INSC, SLCO2B1, PEBP1, DRD3, LRP2, AQP1, DUOX2, CLCA4, ATP6V1B1, ACPP, NPY1R, ACPP*
cellular component	cell projection part	GO:0044463	0.0475	C=639; O=11; E=4.83; R=2.28; rawP=0.0092	*ROBO2, FGF13, PLA2G4F, PEBP1, LRP2, AQP1, DLG2, NLGN1, NCAM1, CTNND2, EVC2*
cellular component	plasma membrane	GO:0005886	0.0193	C=4289; O=47; E=32.44; R=1.45; rawP=0.0028	*SLC22A3, ROBO2, FGF13, GRB14, LRP2, AQP1, FGG, STX1B, PTPRG, ATP6V1B1, NPY1R, ACPP, LY6G6D, NMUR2, GABRP, DRD3, NOTCH3, NCAM1, CPE, UCHL1, ENPP3, SLCO2B1, CNTN5, TRPM8, SLC14A1, CACNA1E, DUOXA1, GPR144, NLGN1, EVC2, EPHA10, PLA2G4F, ITGA7, CHRM5, RHBDL1, GPR133, DLG2, CD207, DSG1, MUC12, DUOX2, SH3TC2, PKP1, CLCA4, CACNA2D1, SLC4A11, BMPR1B*
cellular component	plasma membrane part	GO:0044459	0.0017	C=1918; O=30; E=14.51; R=2.07; rawP=7.80e-05	*SLCO2B1, SLC22A3, ROBO2, TRPM8, SLC14A1, CACNA1E, LRP2, AQP1, NLGN1, FGG, STX1B, PTPRG, ATP6V1B1, NPY1R, EVC2, EPHA10, PLA2G4F, ITGA7, CHRM5, RHBDL1,*
cellular component	apical plasma membrane	GO:0016324	0.0406	C=224; O=6; E=1.69; R=3.54; rawP=0.0072	*SLCO2B1, DUOX2, CLCA4, ATP6V1B1, LRP2, AQP1*
cellular component	secretory granule	GO:0030141	0.0211	C=257; O=7; E=1.94; R=3.60; rawP=0.0034	*KLK13, KLK7, FGG, KLK5, CPE, SCG3, ACPP*
cellular component	extracellular region	GO:0005576	0.0001	C=2140; O=36; E=16.19; R=2.22; rawP=2.20e-06	*CDCP2, KLK10, CDSN, MGP, PEBP1, LRP2, AQP1, FGG, MXRA5, ACPP, EFEMP1, ABI3BP,*
cellular component	extracellular matrix	GO:0031012	0.0142	C=426; O=10; E=3.22; R=3.10; rawP=0.0015	*EFEMP1, LAMA4, ADAMTS5, ABI3BP, MGP, COL5A3, FBLN7, PRELP, MAMDC2, ADAMTS16*
cellular component	extracellular region part	GO:0044421	0.0142	C=1099; O=18; E=8.31; R=2.17; rawP=0.0016	*LAMA4, KLK5, MGP, PEBP1, LRP2, AQP1, KLK13, FGG, PRELP, EFEMP1, ADAMTS5*
cellular component	proteinaceous extracellular matrix	GO:0005578	0.0062	C=360; O=10; E=2.72; R=3.67; rawP=0.0004	*EFEMP1, LAMA4, ADAMTS5, ABI3BP, MGP, COL5A3, FBLN7, PRELP, MAMDC2, ADAMTS16*

Churra *vs.* Assaf. Upregulated Assaf					
**Database**	**Name**	**ID**	**adjP**	**Statistics**	**Genes**
biological process	immune system process	GO:0002376	0.0030	C=1792; O=14; E=4.65; R=3.01; rawP=8.95e-05	*GPR183, AZGP1, ICAM4, IL18BP, HAMP, CCL4, IL36A, IL5RA, MMP1, CD70, APOA4, CSF2, IGLL1, PRSS2*
biological process	immune response	GO:0006955	0.0001	C=1071; O=13; E=2.78; R=4.67; rawP=1.55e-06	*GPR183, AZGP1, ICAM4, IL18BP, HAMP, CCL4, IL36A, IL5RA, CD70, APOA4, CSF2, IGLL1, PRSS2*
biological process	response to stress	GO:0006950	0.0385	C=2952; O=16; E=7.67; R=2.09; rawP=0.0017	*TFPI2, IGSF10, IL5RA, RYR2, MMP1, TRH, TP53I11, PRSS2, GNA14, HEY2, CCL4, HAMP, IL18BP, IL36A, MMP13, APOA4*
molecular function	receptor binding	GO:0005102	0.0010	C=1211; O=11; E=2.85; R=3.86; rawP=6.95e-05	*ICAM4, IL18BP, HAMP, CCL4, IL36A, TRH, CD70, MMP13, CSF2, FGL2, GNA14*
cellular component	extracellular region	GO:0005576	3.75e-05	C=2140; O=18; E=5.40; R=3.34; rawP=1.50e-06	*TFPI2, IGSF10, IL5RA, MMP1, TRH, CSF2, PRSS2, AZGP1, ICAM4, CCL4, HAMP, IL18BP, IL36A, CD70, MMP13, APOA4, IGLL1, FGL2*
cellular component	extracellular region part	GO:0044421	0.0001	C=1099; O=12; E=2.77; R=4.33; rawP=1.15e-05	*TFPI2, IL18BP, CCL4, IL36A, IL5RA, MMP1, CD70, APOA4, MMP13, CSF2, PRSS2, FGL2*
cellular component	extracellular space	GO:0005615	0.0003	C=856; O=10; E=2.16; R=4.63; rawP=4.07e-05	*IL18BP, CCL4, IL36A, IL5RA, CD70, APOA4, MMP13, CSF2, PRSS2, FGL2*
